# The Plant Growth-Promoting Fungus MF23 (*Mycena* sp.) Increases Production of *Dendrobium officinale* (Orchidaceae) by Affecting Nitrogen Uptake and ${\mathrm{NH}}_{4}^{+}$ Assimilation

**DOI:** 10.3389/fpls.2021.693561

**Published:** 2021-07-15

**Authors:** Tingting Shan, Lisi Zhou, Bing Li, Xiaomei Chen, Shunxing Guo, Airong Wang, Lixia Tian, Jingting Liu

**Affiliations:** Institute of Medicinal Plant Development, Chinese Academy of Medical Sciences and Peking Union Medical College, Beijing, China

**Keywords:** *Dendrobium officinale*, *Mycena*, nitrogen uptake, metabolome, glutamate dehydrogenase

## Abstract

*Dendrobium officinale* Kimura et Migo is a traditional and scarce medicinal orchid in China. Mycorrhizal fungi could supply nitrogen (N) to orchids for seed germination and seedling recruitment. However, the N transport mechanism between orchids and the fungus is poorly understand. Early studies found that the fungus MF23 (*Mycena* sp.) could promote the growth of *D. officinale*. To better dissect the molecular interactions involved in N transport between *D. officinale* and MF23, transcriptome and metabolome analyses were conducted on conventional and mycorrhizal cultivations of *D. officinale*. Moreover, validation tests were carried out in the greenhouse to measure net fluxes of $NO_{3}^{-}$ and $NH_{4}^{+}$ of roots by a non-invasive micro-test technology (NMT), determine N assimilation enzyme activity by the ELISA, and analyze the expression level of differentially expressed genes (DEGs) of N transporters and DEGs involved in N metabolism by RT-qPCR. Combined transcriptome and metabolome analyses showed that MF23 may influence N metabolism in *D. officinale*. The expression of *DoNAR2.1* (nitrate transporter-activating protein), *DoAMT11* (ammonium transporter), *DoATFs* (amino acid transporters), *DoOPTs* (oligopeptide transporters), and *DoGDHs* (glutamate dehydrogenases) in symbiotic *D. officinale* was upregulated. NMT results showed a preference for $NH_{4}^{+}$ in *D. officinale* and indicated that MF23 could promote the uptake of $NO_{3}^{-}$ and$NH_{4}^{+}$, especially for $NH_{4}^{+}$. ELISA results showed that MF23 could increase the activity of glutamine synthetase (GS) and glutamate dehydrogenase. This study suggested that MF23 increases the production of *D. officinale* by affecting N uptake and $NH_{4}^{+}$ assimilation capacity.

## Introduction

*Dendrobium officinale* Kimura et Migo (Orchidaceae) is the only plant in Tiepishihu, which has the effects of nourishing Yin, clearing heat, benefiting the stomach, and increasing saliva and has been used as a Chinese traditional medicine and health food for more than 2,000 years (Pharmacopoeia Commission, 2015). The wild resources of *D. officinale* are on the verge of exhaustion because of its low seed germination rate, low growth tendency, and the excessive demand. More than 90% of the medicinal materials in China depend on facility cultivation, with a planting area of nearly 10,000 ha, mainly distributed in Yunnan and Zhejiang (Si et al., 2017a). The industrial scale of *D. officinale* has reached 10 billion RMB, and it has been in constant expansion. However, there are some problems in planting, such as the long growth cycle, high cost, and low nutrient conversion rate. Orchid plants generally have a symbiotic relationship with fungi. Numerous studies have shown that mycorrhiza can have positive effects on orchid plant growth. Mycorrhizal fungi can promote tiller growth and increase the total biomass of *D. officinale* (Tian et al., 2018; Wei et al., 2018; Wang and Hu, 2019; Zhang et al., 2020a). *Ceratobasidium* sp. AR2 inoculation greatly enhanced the fresh weight, dry weight, plant height, root number, and bud number of *Anoectochilus roxburghii* (Zhang et al., 2020b). Fungal strains isolated from the roots of natural *Cymbidium* plants increased the fresh weight, plant height, leaf number, and root length of *Cymbidium goeringii* (Wu et al., 2010). Therefore, the fungus has substantial potential to solve the planting problem of *D. officinale*. In our previous studies, the fungus MF23 (*Mycena* sp.) was shown to promote the growth of *D. officinale* in different regions, improving the root, stem, and leaf weight of *D. officinale* (Chen et al., 2016, 2017; Tian et al., 2018). However, the promoting mechanism is still unclear.

Mycorrhizal fungi supply plants with water and nutrients, such as carbon (C), nitrogen (N), and phosphorus (P), or produce signal molecules, such as rhizosphere secretions, fungal secretions, and fungal elicitors, to promote plant growth (Xu and Zhang, 2007). Among these, N is an essential nutrient for plant growth and the fourth most abundant element in plants (Biswas et al., 2021). Limited N availability adversely affects plant growth and causes serious effects on crop production. N is a major nutrient transferred by mycorrhiza, especially in orchids (Stöckel et al., 2014). Mycorrhiza makes the N concentrations in orchid tissues higher than surrounding autotrophic plants (Hynson et al., 2013). Mycorrhizal fungi supply both inorganic and organic N to orchids for seed germination and seedling recruitment (Cameron et al., 2006; Tedersoo et al., 2007; Yang et al., 2018). At present, the N transport model between arbuscular mycorrhizal fungi (AMF) and plants has been established (Govindarajulu et al., 2005; Jin et al., 2005), in this respect, research on N nutrition transport between orchids and mycorrhizal is not as well studied.

To this end, transcriptome and metabolome analyses were conducted to decipher the mechanism of the interaction between *D. officinale* and MF23. The effects of MF23 on growth, N uptake, and metabolism of *D. officinale* were systematically studied, which revealed the promoting mechanism of MF23 from the perspective of N nutrition for the first time. Studies of symbiotic *D. officinale* provide a reliable molecular basis for further investigations of N transport between fungi and orchids. These lay the theoretical foundation for the usage of mycorrhizal fungi in promoting the production of *D. officinale* and protection of endangered medicinal plants.

## Materials and Methods

### Fungal Materials

Mycorrhizal fungus MF23 (*Mycena* sp.) was isolated from the roots of *D. officinale*, it was preserved by the Mycorrhizal Laboratory of Biotechnology Center, Institute of Medicinal Plant Development (Chen et al., 2016). MF23 was activated and amplified on potato dextrose agar in the dark at 25°C for 20 days. Then, fungal hyphae were transferred to solid medium containing wheat bran and saw dust (1:1, v/v) and were incubated in the dark at 25°C for 30 days to generate a solid inoculum.

### Plant Materials

The *D. officinale* orchids used for transcriptome and metabolome analyses were collected from Jiangsu Yicaotang Zhihu Co. Ltd. (Jiangsu, China). Samples were stems of *D. officinale* grown in a plastic shed for 15 months after acclimatizing for 1 year [collection time and organ specified in the Pharmacopeia (Pharmacopoeia Commission, 2015)]. The substrate in the seedbed was bark mixed with a small amount of stone. About 2 g of fresh, solid form inocula of MF23 were placed near the seedlings roots when seedlings were transplanted to the seedbed (mycorrhizal cultivation of *D. officinale*, T; Chen et al., 2016), and seedlings without fungus were conventionally cultivated (CK). For initial inoculum, 1.5 g clump^−1^ of MF23 inocula was used, and 1.5 g clump^−1^ of supplementary inocula was added every 6 months.

The *D. officinale* orchids used for validation tests were collected from a conventional greenhouse (Institute of Medicinal Plant Development, Beijing). Tissue culture seedlings of *D. officinale* (3–5 cm in height) were cultured on solid medium containing pine bark and sawdust (4:1, v/v). Ten seedlings were transplanted to a solid medium, and 0.5 g of fresh, solid form inocula of MF23 were placed near the seedling roots (dual culture, DC). These conditions were the same for the axenic culture group but without the fungus (AC). The cultures were kept in the greenhouse with a 10 h light/14 h dark photoperiod at 24 ± 1°C and an illumination intensity of 1,500 Lx. Roots of AC and DC were collected at different stages, including 4, 10, and 16 weeks after transplanting.

### Growth Index Measurement

For the Omics samples, one clump of *D. officinale* was used as a repetition. Eighteen repetitions were used for each group. Roots, stems, and leave were collected, dried at 60°C to constant weight, and recorded the dry weight of each organ.

For the validated sample, one culture bottle of *D. officinale* was used as a repetition. Six repetitions were used for each group. At each time point, the dry weight of roots, stems, and leaves was measured, as well as the highest root length, stem diameter, and seedling height.

### Transcriptomic Analysis

Total RNA of the stem samples was extracted using the Improved HUAYUEYANG Quick RNA Isolation Kit (Shan et al., 2018). RNA quality analysis, library construction, sequencing, data filtering and mapping, and differential expression analysis were performed by the Novogene Bioinformatics Technology Co. Ltd. (Beijing, China). Briefly, total RNA degradation and contamination were verified by electrophoresis in a 1.0% agarose gel. RNA concentration and purity were assessed using a NanoDrop^™^ 2000 Spectrophotometers (Thermo Fisher, United States). Sequencing libraries were generated using the NEBNext^®^ Ultra^™^ RNA Library Prep Kit for Illumina^®^ (NEB, United States) using 3 μg of total RNA. Clean reads were obtained from the raw data by removing adapter-containing reads, poly-N-containing reads, and low-quality reads. Then, clean reads were mapped to the *Dendrobium catenatum* reference genome^1^ using the HISAT2 v.2.0.4 software (Kim et al., 2015; Si et al., 2017b). GOseq software was used for gene ontology (GO) annotation (Young et al., 2010). Differential expression analysis was performed using the DESeq R package (1.18.0) with a cutoff probability of ≥0.8 and │log_2_Ratio (R2P2CMS/R2P2)│≥1 (Anders and Huber, 2010). Differentially expressed genes (DEGs) were further assigned to the KEGG (Kyoto Encyclopedia of Genes and Genomes) database. KEGG was used to perform pathway analysis. Pathways with a Q-value ≤0.05 were considered significantly enriched pathways (Minoru et al., 2008). Three biological replicates were used for each sample.

### Metabolomic Analysis

Chromatography was performed on an UHPLC-LTQ Orbitrap (Thermo Fisher Scientific, Bremen, Germany), using a BEH C18 column (100 mm × 2.1 mm i.d., 1.7 μm; Waters, Milford, United States). The mobile phase consisted of (A) 0.1% formic acid in water and (B) 0.1% formic acid in acetonitrile. They were eluted with the following gradient: 0–1.5 min: 5–25% B, 1.5–10.0 min: 25–100% B, 10.0–13.0 min: 100% B, and 13.0–13.5 min: 100–5, 5% B. The final solvent composition was maintained for 10min (5% B). The flow rate was 0.40 ml/min, and the injection volume was 3 μl; the column oven was set to 45°C. MS acquisition was performed with positive and negative ionization. The electrospray capillary, injection, and collision voltage were 3.0 kV, 40 V, and 30 eV, respectively. The capillary temperature was 350°C, and carrier gas flow was 45 l/h. The mass range was 50–1,000 m/z, and resolution was 30,000.

All plasma samples were detected by LC-MS to obtain plasma metabolic profiles between the groups, and Chroma TOF software was used to analyze peak figures and obtain raw data. The standardized LC-MS data matrix was imported into SIMCA-P+14.0 (Umetrics, Umeå, Sweden) to conduct multivariate statistical analysis, including principal component analysis (PCA) to observe the overall distribution between the samples and the stability of the whole analysis process and (orthogonal) signal correction partial least squares-discriminant analysis (O)PLS-DA to distinguish the overall differences in metabolic profiles between groups and find differential metabolites between groups. To prevent the model from over-fitting, the quality of the model was examined by seven cycles of reciprocal verification and 200 response sequencing tests. Differential metabolites between groups were screened by (O)PLS-DA and a *t*-test. The metabolites with VIP >1, *p* < 0.01, and fold change >1.5 were screened as differentially expressed metabolites (DEMs). Finally, plasma differential metabolites between two groups were analyzed by the KEGG pathway database and included channel enrichment and interaction network construction.

### Transcriptome and Metabolome Data Set Integration

To assess the potential relevance of quantitative information between mRNA and metabolites, the *D. catenatum* genome was used to link DEGs and DEMs. R language was used to calculate Pearson’s correlation coefficient. KEGG was used to perform pathway analysis of all correlated DEGs/DEMs.

### Measurements of Net Fluxes of $NO_{3}^{-}$ and $NH_{4}^{+}$

Plants were N starved for 7 days using 1/2 Murashige and Skoog (MS) medium without NH_4_NO_3_ and KNO_3_ prior to flux analysis using the non-invasive micro-test technology (NMT Physiolyzer^®^ Younger USA LLC, Amherst, MA, United States; Li et al., 2017). Six healthy roots were used for nitrate (${\mathrm{NO}}_{3}^{-}$) and ammonium (${\mathrm{NH}}_{4}^{+}$) flux analyses in each group. The net ${\mathrm{NH}}_{4}^{+}$ and ${\mathrm{NO}}_{3}^{-}$ fluxes were measured using NMT at the Xuyue (Beijing) Sci. & Tech. Co., Ltd. (Beijing, China).

Briefly, glass micropipettes (Φ4.5 ± 1 μm, XY-CGQ-01) were pre-pulled and salinized in an electrode controller. After finishing preparation, the end of the micropipettes was filled with backfilling solution (${\mathrm{NH}}_{4}^{+}$, 100 mm NH_4_Cl; ${\mathrm{NO}}_{3}^{-}$, 10 mm KNO_3_; Ruan et al., 2016). The remaining portion was filled with 50–80 μm columns of selective liquid ion-exchange cocktails (${\mathrm{NH}}_{4}^{+}$ LIX, XY-SJ-NH_4_; ${\mathrm{NO}}_{3}^{-}$ LIX, XY-SJ-NO_3_). An Ag/AgCl wire microsensor holder (YG003-Y11, Younger USA) was inserted in the back of the electrode to contact the electrolyte solution. YG003-Y11 was used as the reference microsensor. The flux microsensor was calibrated at the start and end time of each test with the calibration liquid (${\mathrm{NH}}_{4}^{+}$ and ${\mathrm{NO}}_{3}^{-}$: 0.05 and 0.5 mm). The flux microsensor with Nernstian slopes greater than 50 mV for ${\mathrm{NH}}_{4}^{+}$ and less than −50 mV for ${\mathrm{NO}}_{3}^{-}$ per 10-fold concentration difference was used for the following measurements. Before analysis, roots were immersed in a Petri dish that contained 10 ml measuring solution (0.1 mm NH_4_NO_3_, 0.1 mm CaCl_2_, and pH 6.0) and equilibrated for 30 min to lower the impact due to environmental changes.

To determine the area along the root axis corresponding with the maximal net ${\mathrm{NH}}_{4}^{+}$ and ${\mathrm{NO}}_{3}^{-}$ influx, an initial measurement was made along the root, followed by around 300 μm walk steps, ranging from 300 to 20,000 μm away from the root apex. Then, nine regions were measured as the distance from the root tip: 300, 500, 800, 1,500, 3,000, 5,000, 7,500, 10,000, and 20,000 μm. ${\mathrm{NH}}_{4}^{+}$ and ${\mathrm{NO}}_{3}^{-}$ fluxes (ca. 5 μm above the root surface) were measured by a reciprocating motion of the ion-selective microelectrode between two positions, in which the vertical movement distance was 30 μm. The ion flux was read every 6 s. The ion flux was recorded at each measurement point for 5 min. Flux data were acquired and analyzed by the imFluxes V2.0 software [imfluxes.com, Xuyue (Beijing) Sci. & Tech. Co., Ltd., Beijing, China].

### Validation of RNA-seq Data by RT-qPCR

Total RNA of the root samples was extracted by the method described above. The primers designed with Primer Premier 6.0 are shown in Supplementary Table S1. A PrimeScript^™^ RT reagent Kit (TaKaRa, Japan) was used for reverse transcription. First, the RT product (1 μl) was diluted with 20 μl ddH_2_O and used as a template. Then, qPCR was performed in a 15 μl reaction mixture containing 7.5 μl of 2 × SYBR^®^ Premix Ex Taq^™^ II (TaKaRa, Japan), 1.5 μl of cDNA template, and 0.3 μl of each gene specific primers. Three biological replicates and three technical replicates were conducted using the LightCycler^®^ 480II RT-PCR System (Roche, Switzerland). The parameters for the reactions were 95°C for 30 s, 40 cycles of 95°C for 5 s, and 60°C for 30s. The cDNA libraries were standardized to housekeeping gene 18S rRNA (Zhang et al., 2012). The 2^−ΔΔCt^ method was used for evaluating gene expression.

### Determination of N Assimilation Enzyme Activities

Nitrate reductase (NR), nitrite reductase (NiR), glutamine synthetase (GS), glutamate synthase (GOGAT), and glutamate dehydrogenase (GDH) activity in the roots was determined by ELISA, according to the manufacturer’s instructions (ELISA kit, Catalog no. CGM-PMCA1-96, Wuhan Colorful Gene Biological Technology, Wuhan, China).

### Statistical Analyses

Data are expressed as the mean ± standard deviation (SD) from at least three independent biological replicates. Significant differences were determined *via* independent sample *t*-test and one-way ANOVA (*p* < 0.05) by the software package SPSS Statistics 17.0 software (SPSS Inc., Chicago, IL, United States).

## Results

### Sample Information for the Transcriptome and Metabolome

**Figure 1:**
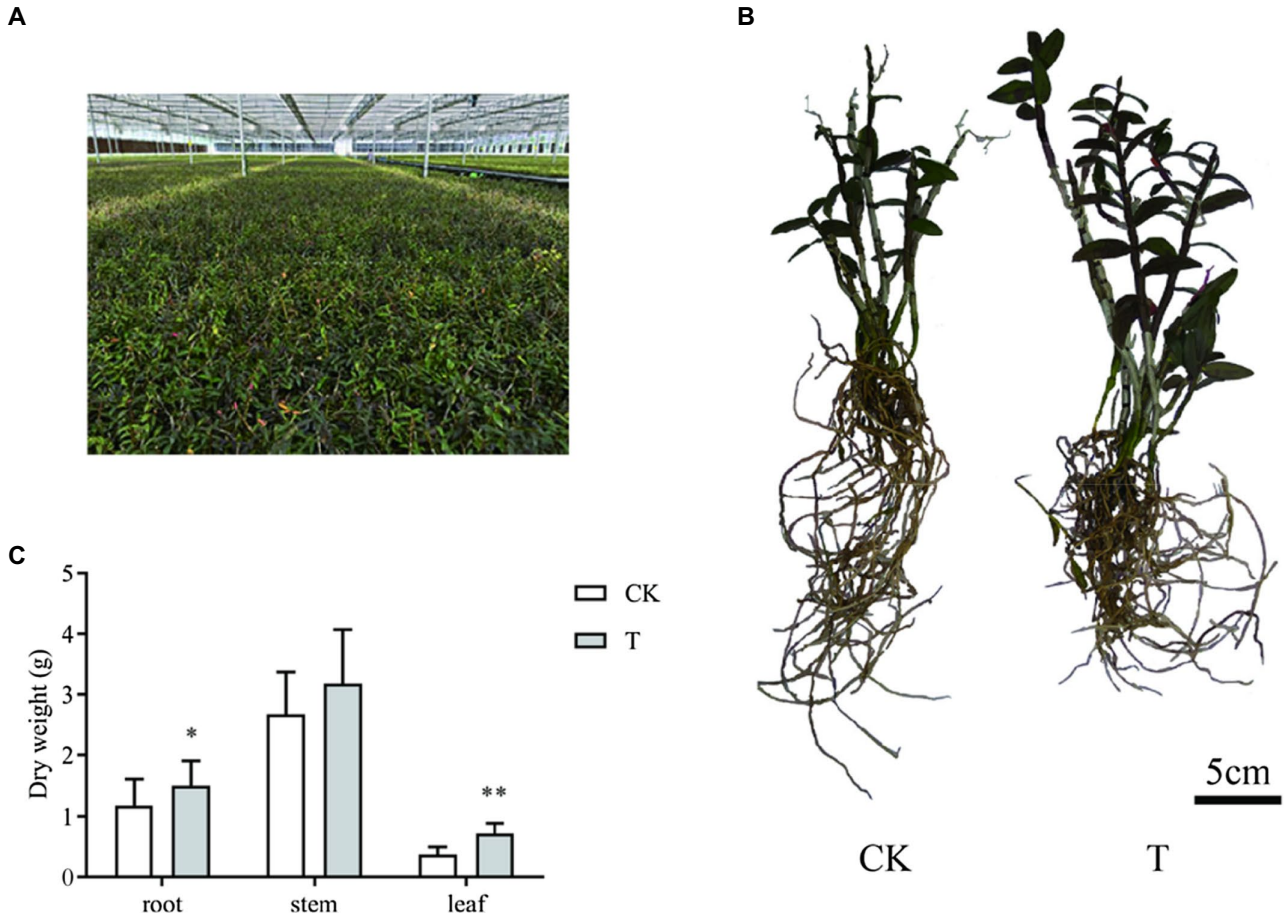
Sample information for transcriptome and metabolome analyses. **(A)** Growth conditions. **(B)** Growth morphology. **(C)** Growth index. CK: conventional cultivation of *Dendrobium officinale* and T: mycorrhizal cultivation of *D. officinale*. ^*^p < 0.05; ^**^p < 0.01.

Transcriptome and metabolome analyses were conducted on conventional and mycorrhizal cultivated *D. officinale* at harvesting time (Figures 1A,B). Mycorrhizal cultivation increased root and leaf dry weight (Figure 1C). The root and leaf dry weight of T was 1.51 ± 0.40 and 0.71 ± 0.18 g, respectively, and was 27.4 and 92.7% higher than CK'.

### Transcriptomic Profiles

In this study, transcriptomic analysis was carried out between T and CK to understand the molecular interactions between *D. officinale* and MF23. In this analysis, 299,481,298 raw reads and 289,048,662 clean reads were obtained (Supplementary Table S2). The sequence data are available in BioProject (Accession number: PRJNA524260) of NCBI. Of the 263 identified DEGs, 94 were upregulated and 169 were downregulated. According to GO gene functional classification system, DEGs were classified to biological process (BP), cellular component (CC), and molecular function (MF) categories and their subcategories. Most DEGs were enriched in BP (e.g., DNA metabolic process, DNA integration, and small GTPase-mediated signal transduction) and MF (e.g., GTP binding, guanyl ribonucleotide binding, and guanyl nucleotide binding; Supplementary Figure S1). Additionally, KEGG pathway analysis revealed 60 enriched pathways, of which, seven pathways exhibited significant differences (*p* < 0.01; Supplementary Figure S2). The differential pathways were ribosome (dct03010), ribosome biogenesis in eukaryotes (dct03008), 2-oxocarboxylic acid metabolism (dct01210), alanine, aspartate, and glutamate metabolism (dct00250), glycerophospholipid metabolism (dct00564), glyoxylate and dicarboxylate metabolism (dct00630), and pentose phosphate pathway (PPP; dct00030).

### Metabolomic Profiles

Principal component analysis was performed and metabolites of T and CK were located on the left and right part, indicating there were differences between groups (Supplementary Figure S3). Differential metabolites were screened by (O)PLS-DA and a student’s *t*-test. Of the 144 metabolites identified as DEMs, 84 were upregulated and 60 were downregulated (Supplementary Table S3). DEMs were annotated in the KEGG databases. Additionally, KEGG pathway analysis revealed 41 enriched pathways, of which, four pathways exhibited significant differences (*p* < 0.01; Supplementary Figure S4). The differential pathways were sphingolipid metabolism (dct00600), biosynthesis of amino acids (dct01230), glyoxylate and dicarboxylate metabolism (dct00630), and glutamate metabolism (dct00250).

### Association Analysis of the Transcriptome and Metabolome Point to N Metabolism

**Figure 2:**
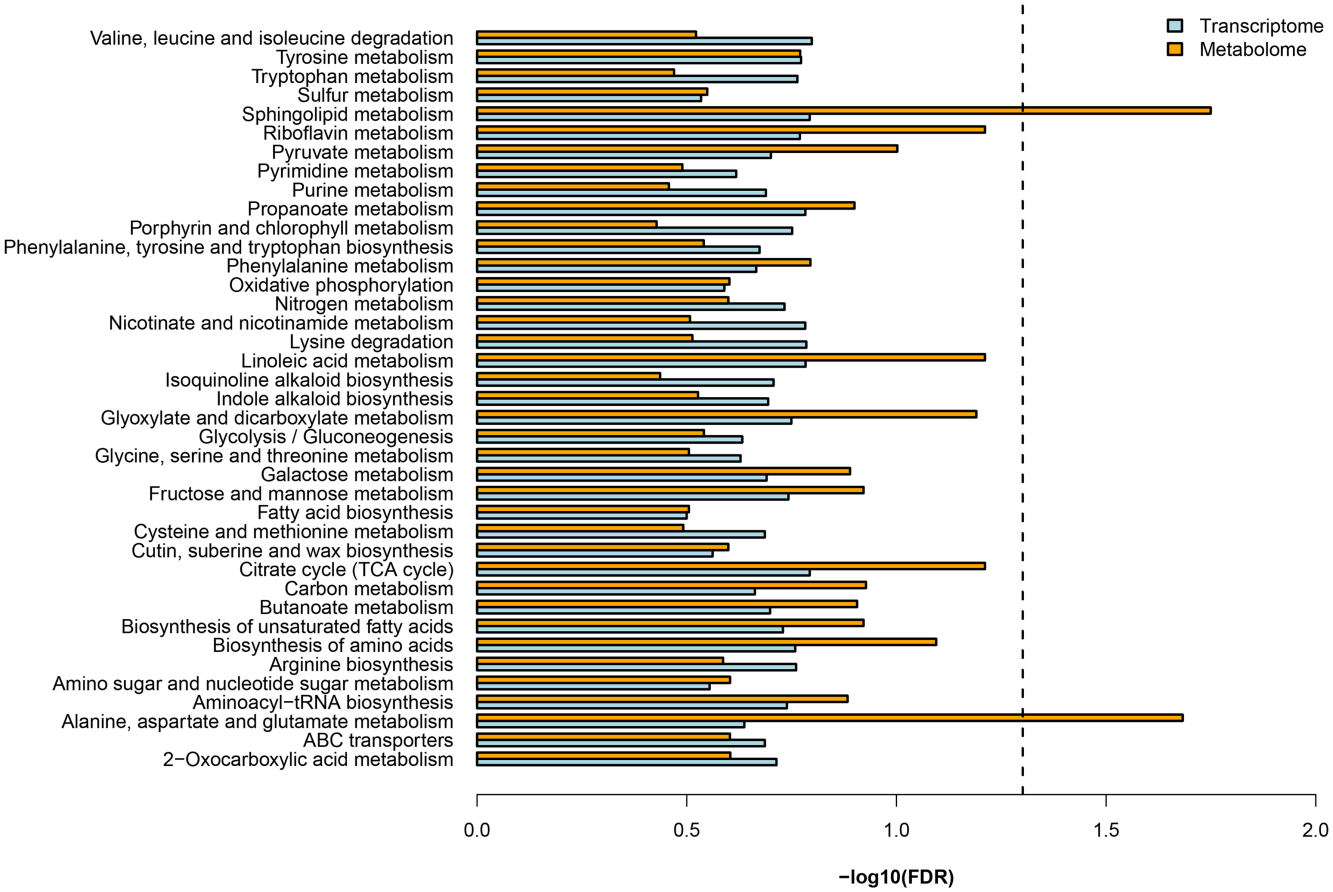
KEGG enrichment analysis of differentially expressed gene (DEGs) and differentially expressed metabolites.

Both DEGs and DEMs were annotated in KEGG databases. KEGG pathway analysis revealed 39 enriched pathways, of which, nitrogen metabolism (dct00910) and several amino acid metabolisms were involved (Figure 2). These results suggested mycorrhizal cultivation could affect N metabolism of *D. officinale*. N uptake by plants is actively regulated by numerous N transporters (Nacry et al., 2013). These transporters display different substrate selectivities. Nitrate transporters (NRTs) function as a ${\mathrm{NO}}_{3}^{-}$ transporter, and ammonium transporters (AMTs) are responsible for the uptake of ${\mathrm{NH}}_{4}^{+}$ (Rennenberg et al., 2010). Transporters that import organic N include amino acid, peptide, and urea transporters (Tegeder and Rentsch, 2010). In the RNA-seq data, nine DEGs of N transporters were found (Table 1). DEGs included two nitrate transporters (NPFs), one nitrate transporter-activating protein (NAR), one amino acid permease (AAP), one lysine/histidine transporter (LHT), one cationic amino acid transporter (CAT), and three oligopeptide transporters (OPTs).

**Table 1:** DEGs of N transporters.

Gene family	Subfamily	Gene ID	Description	Log_2_FC (T/CK)	*P*
NRTs: nitrate transporters	NPF (or NRT1/PTR): nitrate Transporter1/peptide transporter	110109825	Protein NRT1/PTR FAMILY 6.4-like	−3.31	9.86E-05
		110107420	Protein NRT1/ PTR FAMILY 6.4	−2.87	8.10E-03
	NAR: nitrate transporter-activating protein	110104483	High-affinity nitrate transporter-activating protein 2.1-like isoform X1	1.95	2.90E-06
ATF (or AAAP): amino acid transporter family (or amino acid/auxin permease)	AAP: amino acid permease	110111144	Amino acid permease 3-like	−2.40	4.00E-03
	LHT: lysine/histidine transporter	110100062	Lysine histidine transporter-like 8	−1.24	4.00E-03
APC: amino acid-polyamine-choline	CAT: cationic amino acid transporter	110111677	Cationic amino acid transporter 3, mitochondrial-like	−0.91	7.10E-03
OPT: oligopeptide transporter	OPT: oligopeptide transporter	110101252	Oligopeptide transporter 5-like isoform X1	2.80	4.56E10
		110098435	Oligopeptide transporter 1-like isoform X1	0.82	1.46E-02
		110115169	Oligopeptide transporter 4-like	0.65	4.93E-02

N metabolism is a complicated metabolic process mediated by not just N transporters but N assimilation enzymes (Jose and Andrea, 2016). NR and NiR catalyze the further reduction of ${\mathrm{NO}}_{3}^{-}$ to ${\mathrm{NH}}_{4}^{+}$. GS, GOGAT, and GDH allow ${\mathrm{NH}}_{4}^{+}$ to be assimilated into glutamine (Gln). In this study, genes encoding NR were significantly downregulated in mycorrhizal cultivation of *D. officinale*, while genes encoding GDH were significantly upregulated (Table 2). DEMs included Gln (C00064; Supplementary Figure S5), which is the central amino acid linked to ${\mathrm{NH}}_{4}^{+}$ assimilation with amino acid biosynthesis. These results indicated that mycorrhizal cultivation could promote ${\mathrm{NH}}_{4}^{+}$ assimilation into Gln in *D. officinale*.

**Table 2:** DEGs involved in N metabolism.

Description	Enzyme nomenclature	Gene ID	Log_2_FC (T/C)	*P*
*fmdA*, formamidase	[EC:3.5.1.49]	110115292	−2.71	4.80E-02
*NR* [NAD(P)H], nitrate reductase	[EC:1.7.1.1 1.7.1.2 1.7.1.3]	110114643	−1.16	4.63E-02
*GDH* (NADP+), glutamate dehydrogenase	[EC:1.4.1.4]	110091953	1.50	2.58E-02
		110091954	1.64	2.57E-02
*GDH*[NAD(P)+]	[EC:1.4.1.3]	110108713	4.07	4.78E-18

### Sample Information for Validation Tests

**Figure 3:**
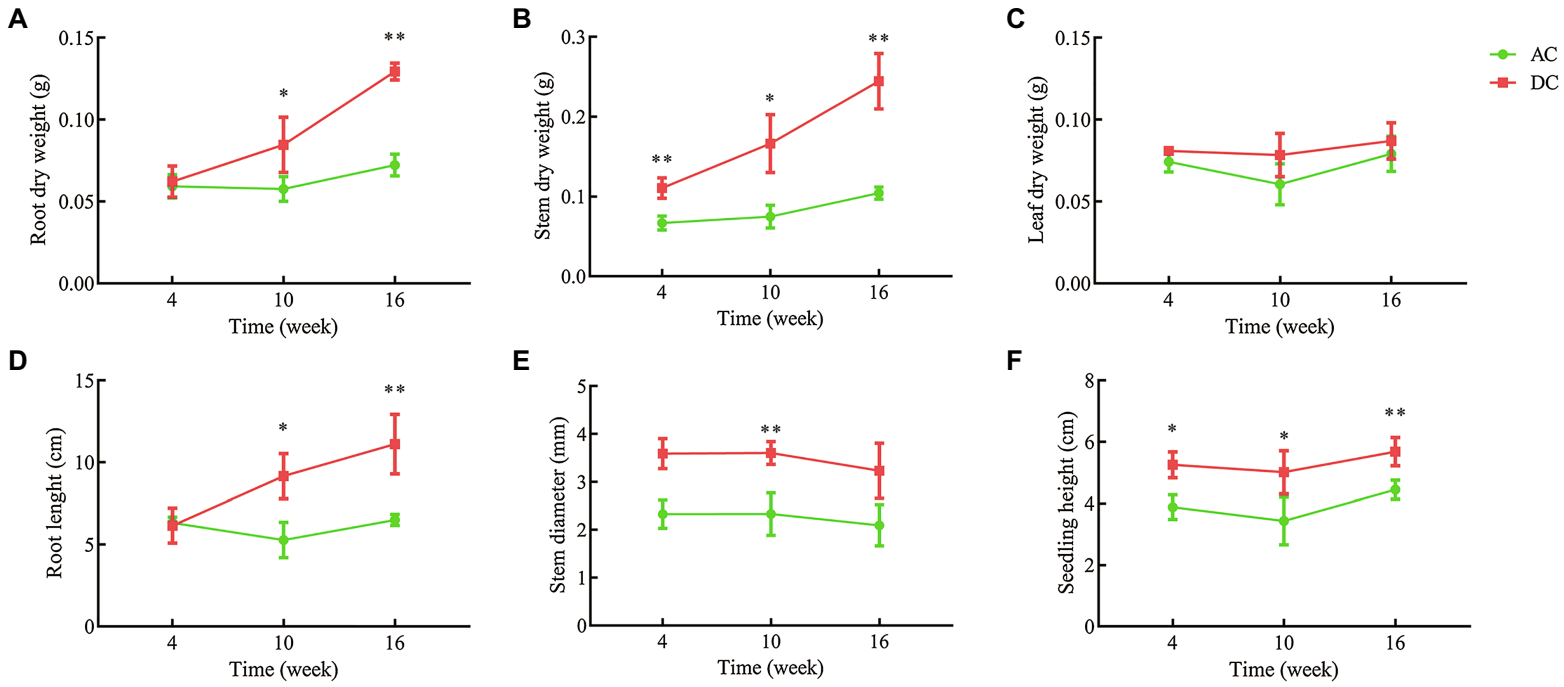
Growth index of samples for validation. **(A)** Root dry weight. **(B)** Stem dry weight. **(C)** Leaf dry weight. **(D)** Root length. **(E)** Stem diameter. **(F)** Seedling height. AC, axenic culture and DC, dual culture. ^*^p < 0.05; ^**^p < 0.01.

Transcriptome and metabolome results indicated that mycorrhizal cultivation could influence N and amino acid metabolism in *D. officinale*. To further study the mechanism of MF23 affecting N metabolism, validation tests were conducted under controllable greenhouse conditions (Supplementary Figures S6, S7). Validation tests showed that MF23 could also increase root and stem dry weight, root length, and seedling height at different growth stages (Figure 3). At week 16, the root and stem dry weight, root length, and seedling height of DC were 0.13 ± 0.01 g, 0.30 ± 0.04 g, 11.12 ± 1.81 cm, and 5.68 ± 0.46 cm, respectively, and were 79.2, 183.5, 71.0, and 27.8% higher than AC', respectively (*p* < 0.01). With the increase of culture time, the effect of MF23 in increasing root and stem dry weight was more significant. Roots are an important plant organ that have multiple functions, including acquisition and fixation of water and nutrients. The stem is the medicinal part of *D. officinale*, as specified in the Pharmacopeia (Pharmacopoeia Commission, 2015). Stem dry weight was closely related to yield and economic benefits.

### Effects of MF23 on $NO_{3}^{-}$ and $NH_{4}^{+}$ Net Fluxes of *D. officinale*

**Figure 4:**
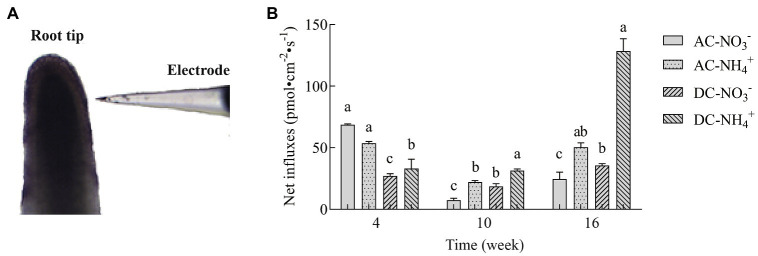
Net influxes of ${\mathrm{NO}}_{3}^{-}$ and ${\mathrm{NH}}_{4}^{+}$ in *D. officinale* (*n* = 6). **(A)** Detected image. **(B)** Effects of fungus MF23 on net ${\mathrm{NO}}_{3}^{-}$ and ${\mathrm{NH}}_{4}^{+}$ influxes in *D. officinale* (*n* = 6). AC, axenic culture and DC, dual culture. The Y-axis represents the maximal net ${\mathrm{NH}}_{4}^{+}$ and ${\mathrm{NO}}_{3}^{-}$ influx. Bars labeled with different letters indicate significant differences between treatments.

NMT is an ion-selective microelectrode technology for the dynamic detection of various ion fluxes of living plants. To determine the effects of MF23 on N uptake in *D. officinale*, NMT was used to analyze the net fluxes of ${\mathrm{NO}}_{3}^{-}$ and ${\mathrm{NH}}_{4}^{+}$ in the roots. As shown in Figure 4, at week 4, ${\mathrm{NO}}_{3}^{-}$ influxes of AC were higher than ${\mathrm{NH}}_{4}^{+}{}^{\prime }$, but AC preferred ${\mathrm{NH}}_{4}^{+}$ to ${\mathrm{NO}}_{3}^{-}$ after that assessment time. The results of ${\mathrm{NO}}_{3}^{-}$ were consistent with ${\mathrm{NH}}_{4}^{+}$ influxes between AC and DC. ${\mathrm{NO}}_{3}^{-}$ and ${\mathrm{NH}}_{4}^{+}$ influxes of DC were lower than AC' at week 4, but higher at week 10 and 16. At week 16, the net influxes of ${\mathrm{NO}}_{3}^{-}$ and ${\mathrm{NH}}_{4}^{+}$ of DC were 35.60 ± 1.49 pmol·cm^−2^·s^−1^ and 128.60 ± 9.83 pmol·cm^−2^·s^−1^, respectively, which were 44.8 and 154.7% higher, respectively, than that in AC. The amount of ${\mathrm{NH}}_{4}^{+}$ was three times that of ${\mathrm{NO}}_{3}^{-}{}^{\prime }$. In conclusion, with the increase in culture time, the effect of MF23 in promoting ${\mathrm{NO}}_{3}^{-}$ and ${\mathrm{NH}}_{4}^{+}$ was more significant, especially in promoting the absorption of ${\mathrm{NH}}_{4}^{+}$.

### Analysis of Transcript Levels of N Transporter DEGs and DEGs Involved in N Metabolism

**Figure 5:**
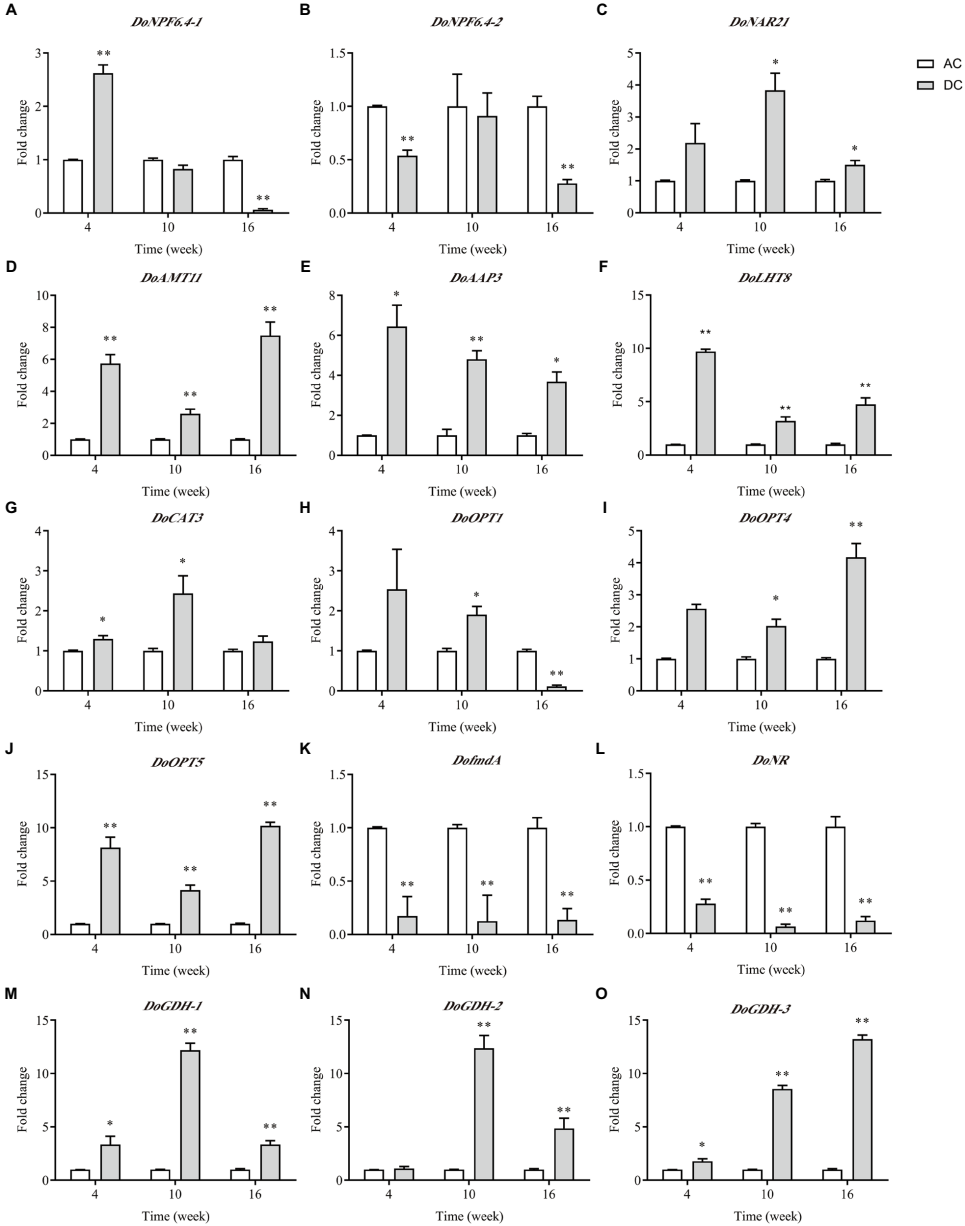
qRT-PCR analysis of DEGs related to N metabolism in *D. officinale* (*n* = 3). **(A–J)**: DEGs of N transporters. **(J–O)**: DEGs involved in N metabolism. The Y-axis represents the mean fold changes in expression values. AC, axenic culture and DC, dual culture. ^*^p < 0.05; ^**^p < 0.01.

Of the nine N transporter DEGs, five DEGs involved in N metabolism, and AMT with minimum *p value* was performed by RT-qPCR analysis (Figure 5). Overall, the expression level of most genes was consistent with the RNA-seq results. Many genes showed high expression in DC, and only *DoNPF6.4-1*, *DoNPF6.4-2*, *DofmdA*, and *DoNR* had low expression at some stages. These downregulated genes were related to ${\mathrm{NO}}_{3}^{-}$ uptake and metabolism. Genes responsible for ${\mathrm{NH}}_{4}^{+}$ uptake and assimilation, such as *DoAMT11* and *DoGDHs*, and genes responsible for amino acid and oligopeptide uptake, such as *DoAAP3, DoLHT8, DoCAT3*, and *DoOPTs*, were upregulated in DC. *DoOPT5* had the highest expression among N transporter genes in DC; the fold change between DC and AC was highest at week 16, which was 10.18 ± 0.35. In contrast to AC, *DoGDHs*’ expression in DC had higher transcript levels. These results indicated that MF23 promotes ${\mathrm{NH}}_{4}^{+}$ and organic N uptake and metabolism.

### Effects of MF23 on Assimilation Enzymes in the N Metabolism of *D. officinale*

**Figure 6:**
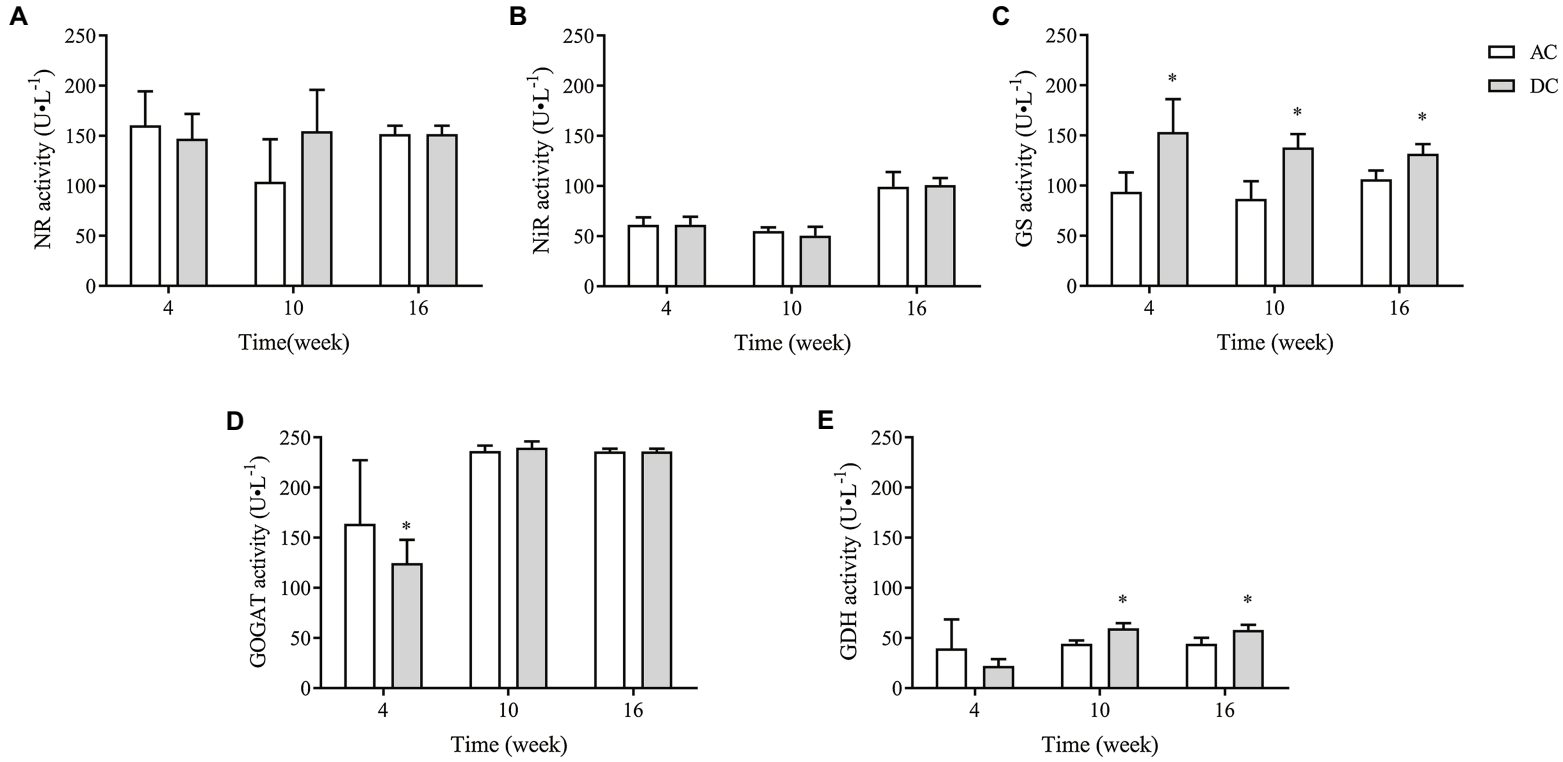
Effects of fungus MF23 on key enzyme activity of nitrogen assimilation in *D. officinale* (*n* = 3). **(A)** Nitrate reductase (NR) activity. **(B)** Nitrite reductase (NiR) activity. **(C)** Glutamine synthetase (GS) activity. **(D)** Glutamate synthase (GOGAT) activity. **(E)** Glutamate dehydrogenase (GDH) activity. AC, axenic culture and DC, dual culture. ^*^p < 0.05; ^**^p < 0.01.

NR, NiR, GS, GOGAT, and GDH were critical rate-limiting enzymes in N metabolism. MF23 increased the activity of GS and GDH in *D. officinale*. At week 10, GS and GDH activity in DC was 137.92 ± 13.61 and 59.74 ± 5.08 U·L^−1^, respectively, which was 58.57 and 35.14% higher than that in AC. At week 16, GS and GDH activity in DC was 131.72 ± 9.84 and (58.17 ± 5.15) U·L^−1^, respectively, which was 23.67 and 31.21% higher than that in AC (*p* < 0.05; Figure 6). These results showed that MF23 promotes ${\mathrm{NH}}_{4}^{+}$ assimilation into Gln and improves the ability to convert inorganic N to organic N by increasing GS and GDH activity.

## Discussion

### MF23 Increases N Uptake in *D. officinale*

Mycorrhizal fungi are able to greatly increase ${\mathrm{NO}}_{3}^{-}$ and ${\mathrm{NH}}_{4}^{+}$ uptake in the terrestrial orchid *C. goeringii* (Wu et al., 2013). Recently numerous studies have demonstrated that some *NRTs* and *AMTs* are exclusively expressed in the symbiotic system. *LjAMT2;2* was exclusively expressed in the mycorrhizal roots of *Cynodon dactylon*, which is responsible for recruiting ${\mathrm{NH}}_{4}^{+}$ and releasing uncharged NH_3_ to the plant cytoplasm (Guether et al., 2009). *NPF* was specifically and preferentially activated in the mycorrhizal roots of rice and was responsible for transporting N compounds between plants and fungi (Drechsler et al., 2017). And, the plant AMT *SvAMT1* and fungal AMT *TcAMT2* were upregulated in the *Tulasnella calospora*–*Serapias vomeracea* symbiosis (Fochi et al., 2017a). These studies emphasized the crucial role of fungi for N transporter activation and N transport in plants. Our observations showed that *DoNAR2.1* and *DoAMT11* were upregulated in the roots of *D. officinale* under a symbiotic system, and more direct evidence showed a high increase in the net influxes of ${\mathrm{NO}}_{3}^{-}$ and ${\mathrm{NH}}_{4}^{+}$ in DC. Moreover, MF23 promoted the uptake of ${\mathrm{NO}}_{3}^{-}$ and ${\mathrm{NH}}_{4}^{+}$ in *D. officinale*, and similar results were also obtained for the orchid *C. goeringii* (Wu et al., 2013).

Plants mainly obtain N nutrition by absorbing ${\mathrm{NO}}_{3}^{-}$ and ${\mathrm{NH}}_{4}^{+}$, but organic N is the major source transferred to orchid hosts from fungi (Cameron et al., 2006; Kuga et al., 2014; Ghirardo et al., 2020). In addition, *SvAAPs, SvLHT*, and *SvOPTs* were more abundant in mycorrhizal protocorms (Fochi et al., 2017b). Our studies also showed that *DoAAP3*, *DoLHT8*, *DoCAT3*, and *DoOPTs* were upregulated in the roots of *D. officinale* under a symbiotic system. Among these, *DoOPT5* showed the highest expression, which suggested that it may play an important role in N acquisition in the MF23-*D. officinale* symbiosis. Oligopeptide transport was thought to be the most effective and fastest way for plants to utilize N (Komarova et al., 2008). Therefore, the mechanisms of how MF23 activates the expression of *DoOPT5* should be further investigated.

### MF23 Increases the $NH_{4}^{+}$ Assimilation Capacity in *D. officinale*

**Figure 7:**
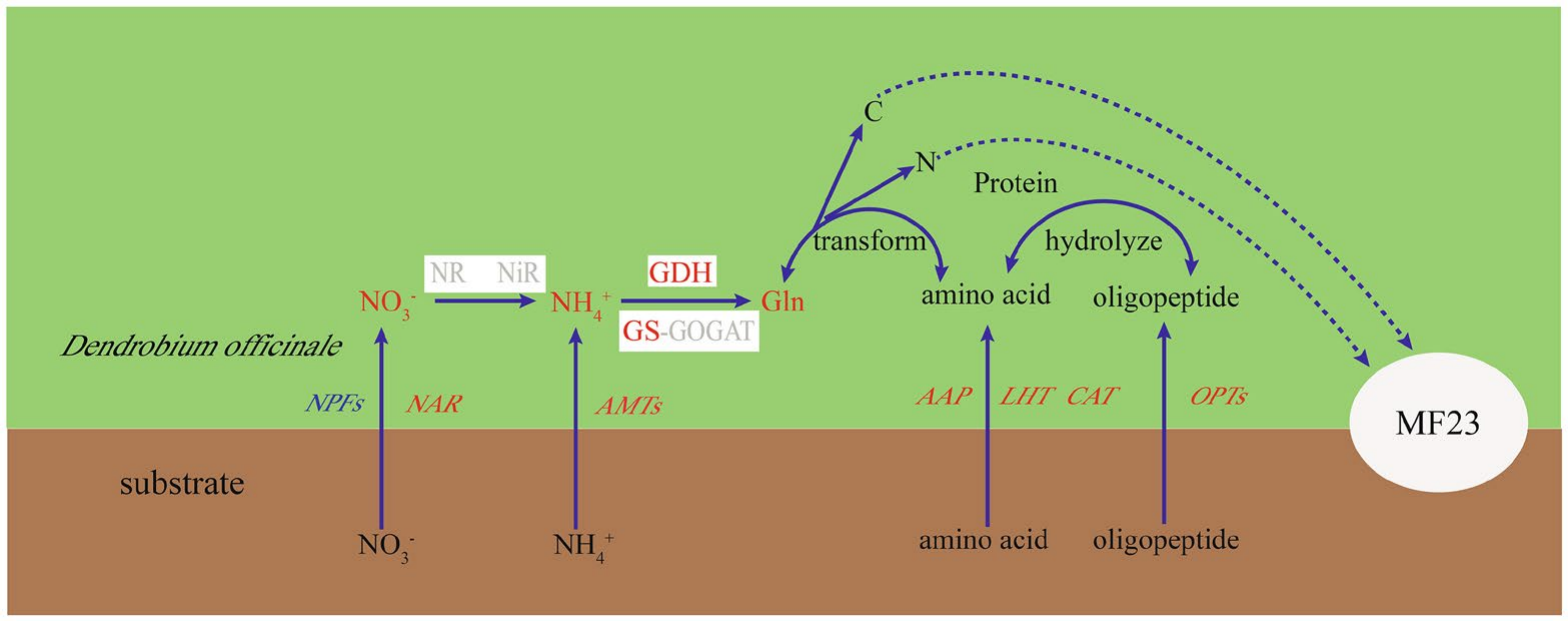
Putative nitrogen uptake and metabolism in the *D. officinale*-MF23 symbiosis. Black font represents the background subjects; red, blue, and gray font represent up-, down-, and undifferentiated-regulated genes or metabolites, respectively. White boxes represent enzymes.

Arbuscular mycorrhizal fungi can absorb ${\mathrm{NO}}_{3}^{-}$ and ${\mathrm{NH}}_{4}^{+}$ from the soil, but ${\mathrm{NO}}_{3}^{-}$ needs to be catalyzed to ${\mathrm{NH}}_{4}^{+}$ first to make it more readily available to plants; this conversion consumes energy (Hawkins et al., 2000). Therefore, extraradical mycelium prefers to absorb ${\mathrm{NH}}_{4}^{+}$ directly and transfer nitrogen to plants (Leigh et al., 2009). Our studies found that *D. officinale* preferred to absorb ${\mathrm{NH}}_{4}^{+}$. The influxes of ${\mathrm{NH}}_{4}^{+}$ were more than twice the net influxes of ${\mathrm{NO}}_{3}^{-}$ at week 16, which was consistent with Zhang et al. (2018), who found that the absorption rate for ${\mathrm{NH}}_{4}^{+}$ was higher than that for ${\mathrm{NO}}_{3}^{-}$ in epiphytic orchids. More interestingly, the function of MF23 to increase influxes of ${\mathrm{NH}}_{4}^{+}$ in *D. officinale* was significantly higher than the increased influxes of ${\mathrm{NO}}_{3}^{-}$. In this study, after dual culture with MF23 for 16 weeks, the increased influxes of ${\mathrm{NH}}_{4}^{+}$ *D. officinale* was three times that of ${\mathrm{NO}}_{3}^{-}{}^{\prime }$. Moreover, the *DoGDHs* were upregulated in RNA-seq data, and the results of RT-qPCR and enzyme activity under symbiotic system were consistent. All results indicated that MF23 increased the ${\mathrm{NH}}_{4}^{+}$ assimilation capacity in *D. officinale*. Based on the results obtained in this study, a schematic map was summarized (Figure 7). MF23 promoted the growth of *D. officinale* by enhancing N uptake and the ${\mathrm{NH}}_{4}^{+}$ assimilation capacity.

### MF23 Influences N Metabolism in *D. officinale* by Regulating C Metabolism

Symbioses between fungi and plants abound in nature. Host plants transfer excess photosynthates to fungi, and in return, fungal partners uptake and transfer soil-derived nutrients and water to the plant. Nutrient exchanges between AMF and host plants are completed by a mycorrhizal symbiosis (Bonfante and Genre, 2010). Li et al. (2014) suggested the transfer and metabolism of C and N in symbioses may play a critical role in maintaining nutrient balance and resource reallocation between the host plant and fungus. C skeletons and energy provided by host plants are used by AMF to transform and transport N; eventually, these N compounds are returned as C donors to host plants (Li et al., 2014). Fellbaum et al. (2012) found that C availability to the mycorrhizal fungus by the host plant may stimulate N uptake and transfer in the fungal partner (Fellbaum et al., 2012). In non-photosynthetic orchid stages, fungal partners provide amino acids to protocorms for supplying C and N sources, and in return, the plant exports ${\mathrm{NH}}_{4}^{+}$ to the fungus (Fochi et al., 2017a). The ornithine cycle of N metabolism is pivotal, linking arginine biosynthesis and C recycling in AMF symbionts (Jin, 2009). These studies suggest that C metabolism is closely associated with N metabolism in mycorrhizal symbioses. In this study, many KEGG significant enrichment pathways were linked to C and N metabolism; for example, alanine, aspartate, and glutamate metabolism belongs to amino acid metabolism. Glyoxylate and dicarboxylate metabolism and the PPP belong to carbohydrate metabolism. Glyoxylate is an intermediate product of photorespiration, which is accompanied by C and N (Younès et al., 2016). PPP is an important pathway for glucose metabolism in plants, and its intermediate product may be involved in the synthesis of amino acids (Huang et al., 2004). Moreover, the results of transcriptome and validation tests showed that *DoGDHs* were upregulated in the roots of *D. officinale* under a symbiotic system. GDH plays a pivotal role in plant C and N metabolism (Miyashita and Good, 2008). MF23 promoted the growth of *D. officinale* by influencing N metabolism associated with C metabolism.

## Conclusion

Transcriptome and metabolome analyses were conducted using conventional and mycorrhizal cultivation of *D. officinale*, which revealed the molecular interactions involved in N metabolism from *D. officinale*’s response to MF23 infection. The results of validation tests indicated that MF23 promotes the production of *D. officinale* by increasing N uptake and ${\mathrm{NH}}_{4}^{+}$ assimilation capacity. These results also demonstrated the importance of organic N transport in the *D. officinale*-MF23 symbiosis. The results of this study laid the foundation for further studies of the molecular mechanisms in orchid-fungus symbioses related to N uptake and metabolism.

## Data Availability Statement

The original contributions presented in the study are publicly available. This data can be found here: NCBI or DDBJ repositories, accession number PRJNA524260 (https://www.ncbi.nlm.nih.gov/bioproject/PRJNA524260).

## Author Contributions

SG and XC designed and directed the entire study. TS performed most of the experiments, analyzed the data, and drafted the manuscript. LZ and JL performed the RT-qPCR analysis. XC, LZ, and LT assisted with sampling and data analysis. XC, LZ, and BL edited the manuscript. All authors have read and approved the final manuscript.

### Conflict of Interest

The authors declare that the research was conducted in the absence of any commercial or financial relationships that could be construed as a potential conflict of interest.

## Footnotes

^1^ https://www.ncbi.nlm.nih.gov/genome/?term=dendrobium+catenatum

## Supplementary Material

Supplementary Figure S1 GO classification of DEGs.

Supplementary Figure S2 KEGG enrichment analysis of DEGs.

Supplementary Figure S3 Principal component analysis (PCA) of conventional and mycorrhizal cultivation of *Dendrobium officinale*.

Supplementary Figure S4 KEGG enrichment analysis of DEMs.

Supplementary Figure S5 KEGG pathway for N metabolism from the transcriptome and metabolome.

Supplementary Figure S6 Growth conditions of samples for validation.

Supplementary Table T1 RT-qPCR primers of N transporter DEGs and DEGs involved in N metabolism.

Supplementary Table T2 Statistical results of sequencing data.

Supplementary Table T3 Differentially expressed metabolites.
