# Supplementary material for: The Plant Growth-Promoting Fungus MF23 (Mycena sp.) Increases Production of Dendrobium officinale (Orchidaceae) by Affecting Nitrogen Uptake and NH4+ Assimilation
Source: Front Plant Sci. 2021 Jul 15;12:693561. doi: 10.3389/fpls.2021.693561 (PMC8451717; doi:10.3389/fpls.2021.693561)
Supplement: Supplementary file 1 [file Data_Sheet_1.zip › Image_7.docx]

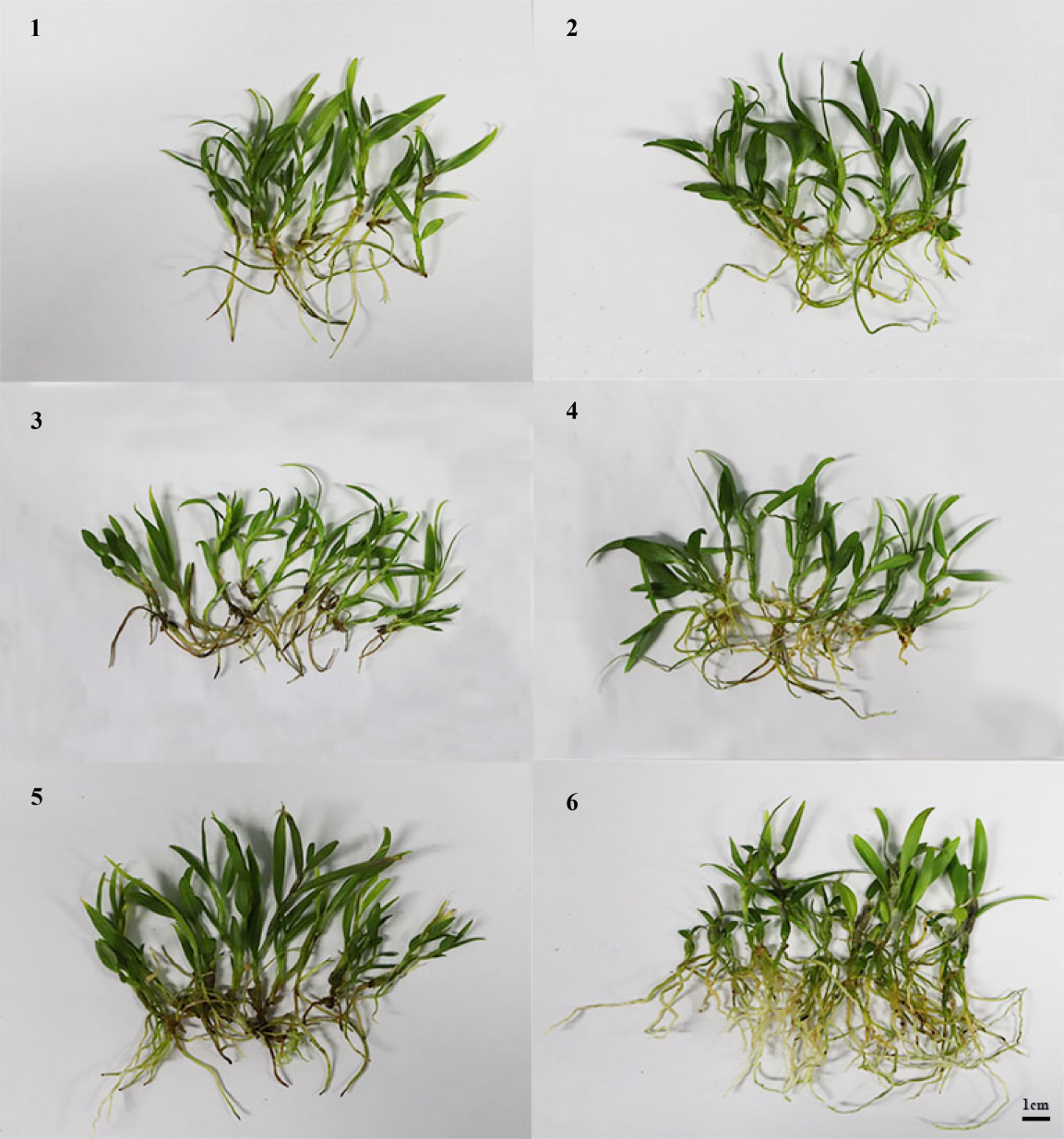


**Supplementary Figure S7.** Growth morphology of samples for validation. (1, 3, 5) represent axenic culture for 4, 10, 16 weeks. (2, 4, 6) represent dual culture for 4, 10, 16 weeks.
