# Supplementary material for: The Plant Growth-Promoting Fungus MF23 (Mycena sp.) Increases Production of Dendrobium officinale (Orchidaceae) by Affecting Nitrogen Uptake and NH4+ Assimilation
Source: Front Plant Sci. 2021 Jul 15;12:693561. doi: 10.3389/fpls.2021.693561 (PMC8451717; doi:10.3389/fpls.2021.693561)
Supplement: Supplementary file 1 [file Data_Sheet_1.zip › Table_1.docx]

**Table S1** RT-qPCR primers of DEGs of nitrogen transporters and DEGs involved in nitrogen metabolism

| **gene** | **gene ID** | **Forward Primer (5'-3')** | **Reverse Primer(3'-5')** |
| --- | --- | --- | --- |
| 18S rRNA |  | TGGTGATGATGCGCCCAGGGCTGTGTT | TCTCGGCCCGCAAGATCCAATCGAAGGA |
| *DoNPF6.4-1* | 110109825 | ATGGTGGCGGCGATGGTGAT | GAAGCTCCTGCACATGCTCTGG |
| *DoNPF6.4-2* | 110107420 | GGCTCGCTCTCCTTCTTCCTCT | GCAGCAACAACCATCGCAATCA |
| *DoNAR21* | 110104483 | AACCAAACCCTCCCTCCGAACA | CCACCGCACCAGAAGAGTCAAC |
| *DoAMT11* | 110099009 | ACGACGGCTGTTGATACGACCT | GGCGACCAGAACCAGTGAGAGA |
| *DoAAP3* | 110111144 | AACGATGTGGACGGCGAGTGCT | ATGACGGCGGCGACAATGGAGA |
| *DoLHT8* | 110100062 | ACGCCGAGGCTGCTTAGTCCAA | AGCCAAGCGTCCTGCGGATTGA |
| *DoCAT3* | 110111677 | AGGCGTTGGCTCCACAATAGGT | GCGGCAATTCCAGCGATCAGAA |
| *DoOPT1* | 110098435 | AGTCGGATGAGGCTTCGGCTAC | AGACCTCCATTCGGCTGCTCTC |
| *DoOPT4* | 110115169 | CGAACGGAGCCTCTTGTCATCA | AAGCGGTGGTCTTCCTTCTCAT |
| *DoOPT5* | 110101252 | CAACTGCAACTTCCGTGGTGGG | GCCCTGGTGCCTGATTTGTCGT |
| *DofmdA* | 110115292 | AGGGCATAAGCGTGGACGAGAG | GCAGCAGGAGAGGAGGAGGTAA |
| *DoNR* | 110114643 | GTGGTGGAGGTGGATGTTCTGG | AGGCGAAGACGAAGGTGAAGAC |
| *DoGDH-1* | 110091953 | CTCTTTCGCAAACCGTCTACCG | TGGAGCACACCTTCCTCATCAA |
| *DoGDH-2* | 110091954 | CGGAGGAGAACAAGCGAGGTAT | CTGAGAAGCTGCGATGGAAGGA |
| *DoGDH-3* | 110108713 | TCCCGTCTCCCTCTCCTTCAGT | TCTTGGAGGCGAAGTCGGATGG |
