# Supplementary material for: The Plant Growth-Promoting Fungus MF23 (Mycena sp.) Increases Production of Dendrobium officinale (Orchidaceae) by Affecting Nitrogen Uptake and NH4+ Assimilation
Source: Front Plant Sci. 2021 Jul 15;12:693561. doi: 10.3389/fpls.2021.693561 (PMC8451717; doi:10.3389/fpls.2021.693561)
Supplement: Supplementary file 1 [file Data_Sheet_1.zip › Table_2.docx]

**Table S2** Statistical result of sequencing data

| **Sample** | **Raw reads** | **Clean reads** | **Clean bases** | **Error rate(%)** | **Q20(%)** | **Q30(%)** | **GC content(%)** |
| --- | --- | --- | --- | --- | --- | --- | --- |
| C-1 | 63924156 | 62035674 | 9.31G | 0.01 | 97.69 | 94.02 | 46.01 |
| C-2 | 50891212 | 48832538 | 7.32G | 0.02 | 95.77 | 89.63 | 46.50 |
| C-3 | 48468998 | 46558552 | 6.98G | 0.02 | 95.87 | 89.84 | 46.38 |
| T-1 | 44998412 | 43326086 | 6.50G | 0.02 | 96.10 | 90.29 | 45.40 |
| T-2 | 40862086 | 39447444 | 5.92G | 0.02 | 96.17 | 90.43 | 45.82 |
| T-3 | 50336434 | 48848368 | 7.33G | 0.01 | 97.65 | 93.91 | 45.84 |
