# Supplementary material for: The Plant Growth-Promoting Fungus MF23 (Mycena sp.) Increases Production of Dendrobium officinale (Orchidaceae) by Affecting Nitrogen Uptake and NH4+ Assimilation
Source: Front Plant Sci. 2021 Jul 15;12:693561. doi: 10.3389/fpls.2021.693561 (PMC8451717; doi:10.3389/fpls.2021.693561)
Supplement: Supplementary file 1 [file Data_Sheet_1.zip › Table_3.docx]

**Table S3** Differentially expressed metabolites

| **Var ID (Primary)** | **m/z** | **r.t(min)** | **metabolites** | **Δppm** | **VIP value** | **FC(T/CK)** | **P** |
| --- | --- | --- | --- | --- | --- | --- | --- |
| 355.0859_5.26 | 355.0859 | 5.26 | 6,7,3'-Trimethoxy-4',5'-methylenedioxyisoflavone | 10 | 2.73928 | 30.21430782 | 6.23112E-08 |
| 383.11563_5.66 | 383.11563 | 5.66 | S-Adenosylhomocysteine | 3 | 5.28628 | 30.06943617 | 1.3711E-08 |
| 301.14362_5.51 | 301.14362 | 5.51 | Nordihydroguaiaretic Acid | 3 | 2.26956 | 29.74340414 | 1.09137E-07 |
| 302.14982_7.12 | 302.14982 | 7.12 | N(6)-[(Indol-3-yl)acetyl]-L-lysine | 3 | 3.04821 | 8.032803435 | 0.001504938 |
| 305.17172_7.21 | 305.17172 | 7.21 | Prosolanapyrone II | 9 | 2.21575 | 6.85964179 | 0.00406336 |
| 301.14889_7.11 | 301.14889 | 7.11 | Nordihydroguaiaretic Acid | 14 | 6.96796 | 6.724381644 | 0.001041158 |
| 305.17596_8.11 | 305.17596 | 8.11 | 12-oxo-14,18-dihydroxy-9Z,13E,15Z-octadecatrienoic acid | 2 | 4.13864 | 5.743624382 | 0.001633021 |
| 303.15911_7.63 | 303.15911 | 7.63 | Prosolanapyrone II | 3 | 10.0676 | 4.684255626 | 0.001096244 |
| 289.14437_5.71 | 289.14437 | 5.71 | Sodium lauryl sulfate | 0 | 2.5201 | 3.706391512 | 8.62897E-10 |
| 323.06031_4.4 | 323.06031 | 4.4 | Grevilline A | 12 | 2.74508 | 3.472961812 | 8.82494E-09 |
| 381.10145_5.48 | 381.10145 | 5.48 | Velloquercetin 4'-methyl ether | 9 | 4.43848 | 2.933635581 | 1.34246E-06 |
| 268.13221_4.87 | 268.13221 | 4.87 | L-Agaritine | 11 | 3.20893 | 2.810660449 | 3.59019E-11 |
| 266.1186_4.99 | 266.1186 | 4.99 | 6-Azaequilenin | 0 | 1.61141 | 2.447043192 | 8.23391E-11 |
| 298.14171_4.95 | 298.14171 | 4.95 | 4-Hydroxypropranolol | 1 | 2.06794 | 2.332510594 | 8.42816E-12 |
| 259.13101_5.67 | 259.13101 | 5.67 | 3'-O-methylbatatasin III | 7 | 2.84414 | 2.23157658 | 3.76974E-08 |
| 241.07652_5.63 | 241.07652 | 5.63 | Lumichrome | 14 | 3.01365 | 1.797839135 | 2.49122E-08 |
| 515.30463_7.96 | 515.30463 | 7.96 | Ganoderic acid A | 6 | 2.09668 | 1.793658325 | 1.42111E-07 |
| 191.02004_0.58 | 191.02004 | 0.58 | Citric acid | 1 | 4.18476 | 1.771752113 | 7.99248E-10 |
| 344.14732_4.41 | 344.14732 | 4.41 | N-trans-Feruloyl-4-O-methyldopamine | 5 | 1.83027 | 1.749127944 | 8.92481E-11 |
| 274.12818_4.56 | 274.12818 | 4.56 | Procainamide | 12 | 2.18122 | 1.645512992 | 1.76185E-07 |
| **Var ID (Primary)** | **m/z** | **r.t(min)** | **metabolites** | **Δppm** | **VIP value** | **FC(T/CK)** | ***P*** |
| 130.06977_0.73 | 130.06977 | 0.73 | 2,5-Dihydro-2,4,5-trimethylthiazole | 9 | 3.23597 | 1.641420071 | 4.15294E-09 |
| 147.07722_0.67 | 147.07722 | 0.67 | L-Glutamine | 5 | 2.71074 | 1.634663109 | 1.49892E-09 |
| 391.24016_5.89 | 391.24016 | 5.89 | Beta-Cortol | 13 | 1.89963 | 1.611650929 | 1.27192E-07 |
| 284.1258_4.27 | 284.1258 | 4.27 | N-(p-Hydroxyphenyl)ethyl p-hydroxycinnamide | 8 | 4.43782 | 1.591943415 | 1.55867E-11 |
| 335.21642_5.88 | 335.21642 | 5.88 | 20-carboxy Arachidonic Acid | 15 | 2.26488 | 1.574185496 | 1.23391E-10 |
| 275.12909_5.27 | 275.12909 | 5.27 | 4,4'-dihydroxy-3,5-dimethoxydihydrostilbene | 4 | 3.196 | 1.563749769 | 2.18202E-05 |
| 287.0905_4.98 | 287.0905 | 4.98 | Dihydrobiochanin A | 3 | 1.56984 | 1.526636622 | 1.23104E-07 |
| 295.22508_5.78 | 295.22508 | 5.78 | 4-hydroxy palmitic acid | 2 | 1.85381 | 1.525911376 | 2.74864E-10 |
| 242.0821_4.64 | 242.0821 | 4.64 | N-Desmethyltolmetin | 0 | 3.42619 | 1.518140538 | 0.000378995 |
| 342.13409_4.49 | 342.13409 | 4.49 | N-trans-Feruloyl-4-O-methyldopamine | 1 | 1.59624 | 1.50680854 | 1.85954E-08 |
| 300.2876_7.07 | 300.2876 | 7.07 | Sphingosine | 7 | 2.79032 | 1.488959576 | 4.54315E-08 |
| 277.21775_9.28 | 277.21775 | 9.28 | γ-Linolenic Acid | 1 | 3.68301 | 1.488929746 | 9.2921E-09 |
| 298.10793_4.1 | 298.10793 | 4.1 | N-cis-Caffeoyltyramine | 1 | 2.03461 | 1.488342364 | 1.34239E-08 |
| 683.2247_0.66 | 683.2247 | 0.66 | Lactulose | 3 | 4.35299 | 1.482290098 | 4.46111E-09 |
| 407.23804_5.35 | 407.23804 | 5.35 | Annoglabasin C | 11 | 1.99803 | 1.473803824 | 3.94238E-08 |
| 282.27687_7.08 | 282.27687 | 7.08 | Oleamide | 8 | 2.13883 | 1.456052085 | 5.91792E-07 |
| 273.10994_5.58 | 273.10994 | 5.58 | 3',4'-Dihydroxy-7-methoxyflavan | 8 | 2.74759 | 1.452894003 | 1.33026E-09 |
| 270.27753_7.2 | 270.27753 | 7.2 | C17 Sphinganine | 7 | 1.80509 | 1.42835109 | 1.11395E-07 |
| 293.21227_6.53 | 293.21227 | 6.53 | alpha-kamlolenic acid | 0 | 2.64117 | 1.427642934 | 4.07885E-08 |
| 283.11558_4.34 | 283.11558 | 4.34 | 2-Phenylethyl beta-D-glucopyranoside | 11 | 2.19626 | 1.426209926 | 8.34119E-09 |
| 285.07439_5.44 | 285.07439 | 5.44 | Biochanin A | 4 | 2.69708 | 1.42370954 | 1.32266E-05 |
| **Var ID (Primary)** | **m/z** | **r.t(min)** | **metabolites** | **Δppm** | **VIP value** | **FC(T/CK)** | ***P*** |
| 287.07234_4.46 | 287.07234 | 4.46 | Irenolone | 3 | 2.34986 | 1.418105733 | 4.89379E-06 |
| 309.20716_5.66 | 309.20716 | 5.66 | 9-hydroperoxy-10E,12,15Z-octadecatrienoic acid | 0 | 2.19412 | 1.418058666 | 5.47701E-07 |
| 281.24891_11.14 | 281.24891 | 11.14 | Oleic Acid | 1 | 2.26556 | 1.414585821 | 6.70624E-06 |
| 288.2873_7.21 | 288.2873 | 7.21 | C17 Sphinganine | 8 | 2.69394 | 1.409805692 | 6.69498E-08 |
| 243.10005_5.08 | 243.10005 | 5.08 | Encelin | 10 | 1.86578 | 1.40316858 | 4.62962E-09 |
| 302.30374_8.05 | 302.30374 | 8.05 | Sphinganine | 5 | 4.57154 | 1.400143008 | 7.84516E-06 |
| 284.29235_8.05 | 284.29235 | 8.05 | Stearamide | 6 | 2.92627 | 1.395105266 | 8.3599E-06 |
| 318.29733_7.08 | 318.29733 | 7.08 | Phytosphingosine | 9 | 8.1729 | 1.391959309 | 1.41435E-08 |
| 401.15713_4.56 | 401.15713 | 4.56 | (+)-Echinoisoflavanone | 5 | 2.09126 | 1.389081131 | 1.34534E-05 |
| 291.19608_6.79 | 291.19608 | 6.79 | alpha-licanic acid | 1 | 1.519 | 1.380054747 | 3.4332E-07 |
| 328.11875_4.19 | 328.11875 | 4.19 | N-trans-Feruloyloctopamine | 0 | 1.78875 | 1.374082954 | 3.64079E-08 |
| 282.1139_4.34 | 282.1139 | 4.34 | (S)-3'-Hydroxycoclaurine | 3 | 4.87723 | 1.37160969 | 3.52206E-08 |
| 311.22242_6.01 | 311.22242 | 6.01 | 9-hydroperoxy-10E,12-octadecadienoic acid | 1 | 3.23714 | 1.370045241 | 1.99716E-07 |
| 353.06907_4.94 | 353.06907 | 4.94 | Quercetin 3-isobutyrate | 8 | 1.73848 | 1.368176113 | 0.001113292 |
| 314.1363_4.34 | 314.1363 | 4.34 | Ethyl N-benzoyl-L-tyrosinate | 7 | 6.93728 | 1.367359505 | 4.03901E-09 |
| 537.30012_6.78 | 537.30012 | 6.78 | Coroglaucigenin-3-o-alpha-L-rhamnopyranoside | 10 | 2.39072 | 1.356816012 | 1.15605E-06 |
| 315.13849_4.34 | 315.13849 | 4.34 | [6]-Shogaol | 8 | 3.04017 | 1.351098495 | 1.3269E-09 |
| 295.22794_7.08 | 295.22794 | 7.08 | Dimorphecolic acid | 0 | 4.47567 | 1.343254413 | 9.40101E-07 |
| 271.09675_5.7 | 271.09675 | 5.7 | (+)-Vestitol | 3 | 4.04685 | 1.341481717 | 6.76211E-10 |
| 295.22508_7.13 | 295.22508 | 7.13 | 4-hydroxy palmitic acid | 2 | 2.02639 | 1.33684839 | 9.33352E-08 |
| 273.11329_5.42 | 273.11329 | 5.42 | 4,4'-dihydroxy-3,5-dimethoxydihydrostilbene | 0 | 5.09862 | 1.329545293 | 5.58364E-05 |
| **Var ID (Primary)** | **m/z** | **r.t(min)** | **metabolites** | **Δppm** | **VIP value** | **FC(T/CK)** | ***P*** |
| 303.12316_5.1 | 303.12316 | 5.1 | Linifolin A | 2 | 2.2284 | 1.327646206 | 0.00019833 |
| 289.10767_4.64 | 289.10767 | 4.64 | 5,6-Dihydro-11-methoxyyangonin | 1 | 2.80672 | 1.327644669 | 0.000108102 |
| 279.2331_10.15 | 279.2331 | 10.15 | Linoleic acid | 0 | 5.90665 | 1.306884304 | 4.87635E-07 |
| 469.1824_5.02 | 469.1824 | 5.02 | Artoindonesianin B | 7 | 2.20894 | 1.297360894 | 4.14717E-05 |
| 565.15021_3.27 | 565.15021 | 3.27 | Pelargonidin 3-lathyroside | 8 | 2.10413 | 1.288758001 | 2.93775E-06 |
| 273.07724_4.75 | 273.07724 | 4.75 | Phloretin | 1 | 2.20555 | 1.287546966 | 4.77229E-06 |
| 395.19019_3.32 | 395.19019 | 3.32 | Zwittermicin A | 1 | 2.68171 | 1.287538752 | 5.41701E-07 |
| 121.06396_5.29 | 121.06396 | 5.29 | p-Tolualdehyde | 6 | 1.64395 | 1.280240013 | 0.000694438 |
| 541.15284_5.74 | 541.15284 | 5.74 | 5''-(4-Hydroxy-(E)-cinnamoyl) alpha-L-arabinofuranosyl-(1->3)-beta-D-xylopyranosyl-(1->4)-D-xylopyranoside | 5 | 1.61613 | 1.278128829 | 0.000285017 |
| 259.09711_4.64 | 259.09711 | 4.64 | 8-Deoxylactucin | 1 | 4.94479 | 1.265440999 | 0.000998963 |
| 193.08502_5.03 | 193.08502 | 5.03 | cis-1,2-Dihydroxy-1,2-dihydro-7-hydroxymethylnaphthalene | 4 | 2.08322 | 1.259969155 | 8.54733E-06 |
| 188.06972_2.29 | 188.06972 | 2.29 | Dl-Indole-3-lactic acid | 5 | 3.05613 | 1.259499456 | 2.22497E-08 |
| 393.2596_6.33 | 393.2596 | 6.33 | Acetoxy-10-gingerol | 10 | 2.19546 | 1.259262596 | 3.50784E-06 |
| 273.09286_4.56 | 273.09286 | 4.56 | 5-S-Cysteinyldopamine | 9 | 2.3399 | 1.252832257 | 0.000122194 |
| 353.26521_6.79 | 353.26521 | 6.79 | Ceriporic acid C | 9 | 2.0848 | 1.246740808 | 3.02955E-06 |
| 563.13929_3.37 | 563.13929 | 3.37 | Pelargonidin 3-lathyroside | 2 | 1.90413 | 1.24443235 | 2.62214E-05 |
| 205.09552_2.28 | 205.09552 | 2.28 | L-Tryptophan | 6 | 2.19516 | 1.235166847 | 2.59847E-07 |
| 329.23354_4.93 | 329.23354 | 4.93 | 11,12,13-trihydroxy-9-octadecenoic acid | 0 | 2.67283 | 1.232861711 | 1.84003E-05 |
| 593.26968_9.81 | 593.26968 | 9.81 | PI(16:1(9Z)/0:0) | 0 | 3.17117 | 1.224644443 | 8.35522E-06 |
| 467.17007_5.14 | 467.17007 | 5.14 | 2',4',4-Trihydroxy-3'-prenylchalcone 4'-O-glucoside | 1 | 2.81605 | 1.219635037 | 0.000171591 |
| **Var ID (Primary)** | **m/z** | **r.t(min)** | **metabolites** | **Δppm** | **VIP value** | **FC(T/CK)** | ***P*** |
| 327.21757_4.76 | 327.21757 | 4.76 | 9,12,13-trihydroxy-10,15-octadecadienoic acid | 0 | 1.85115 | 1.207545189 | 9.39666E-05 |
| 312.12311_4.43 | 312.12311 | 4.43 | Acetylcaranine | 3 | 5.49202 | 1.207437065 | 1.34474E-06 |
| 171.0647_1.54 | 171.0647 | 1.54 | DL-3,4-Dihydroxyphenyl glycol | 2 | 1.81854 | 0.818113363 | 2.67331E-08 |
| 385.1619_2.9 | 385.1619 | 2.9 | Ursiniolide A | 8 | 1.85631 | 0.816577589 | 1.00546E-05 |
| 609.2661_9.53 | 609.2661 | 9.53 | (3Z)-Phycoerythrobilin | 3 | 2.12124 | 0.816412781 | 5.26989E-05 |
| 298.09522_2.25 | 298.09522 | 2.25 | 5-Deoxy-5'-methylthioadenosine | 3 | 2.12433 | 0.813608621 | 1.77121E-07 |
| 463.21867_3.78 | 463.21867 | 3.78 | Dulciol B | 13 | 2.39406 | 0.812967924 | 1.8765E-07 |
| 417.13999_3.14 | 417.13999 | 3.14 | Oleoside dimethyl ester | 0 | 2.44992 | 0.805969015 | 1.00663E-06 |
| 581.22315_3.82 | 581.22315 | 3.82 | Carvedilol glucuronide | 15 | 2.08004 | 0.797187398 | 3.37123E-07 |
| 565.15052_3.87 | 565.15052 | 3.87 | Pelargonidin 3-lathyroside | 8 | 1.88319 | 0.795062667 | 1.67658E-08 |
| 473.1654_4.2 | 473.1654 | 4.2 | 6-Feruloylglucose 2,3,4-trihydroxy-3-methylbutylglycoside | 2 | 2.60379 | 0.791681414 | 5.8546E-07 |
| 417.16214_4.07 | 417.16214 | 4.07 | Chapelieric acid methyl ester | 19 | 3.05248 | 0.789813829 | 2.11134E-07 |
| 167.07939_1.55 | 167.07939 | 1.55 | Nicotinamide, N-(2-hydroxyethyl)- | 12 | 2.37657 | 0.78980456 | 7.5112E-05 |
| 487.21221_3.92 | 487.21221 | 3.92 | O-Desmethylquinidine glucuronide | 9 | 1.7714 | 0.787716706 | 5.32533E-09 |
| 144.09506_0.7 | 144.09506 | 0.7 | Dimaprit | 6 | 1.78384 | 0.786411165 | 1.43066E-06 |
| 579.20931_4.07 | 579.20931 | 4.07 | (+)-Syringaresinol O-beta-D-glucoside | 1 | 2.93658 | 0.781553122 | 4.75863E-09 |
| 229.15314_0.9 | 229.15314 | 0.9 | Pro-Leu | 5 | 2.90588 | 0.77986978 | 1.64867E-08 |
| 459.1516_4.25 | 459.1516 | 4.25 | Paeonolide | 1 | 4.28581 | 0.778007957 | 7.1297E-08 |
| 421.14706_4.65 | 421.14706 | 4.65 | Afzelechin 3-O-alpha-L-rhamnopyranoside | 5 | 2.91789 | 0.774714831 | 5.00723E-09 |
| 207.05151_0.7 | 207.05151 | 0.7 | (S)-dihydrolipoic acid | 1 | 1.7848 | 0.770564728 | 2.31856E-07 |
| 377.18035_3.83 | 377.18035 | 3.83 | Albafuran A | 11 | 4.18607 | 0.769213433 | 3.54613E-09 |
| **Var ID (Primary)** | **m/z** | **r.t(min)** | **metabolites** | **Δppm** | **VIP value** | **FC(T/CK)** | ***P*** |
| 367.15207_3.86 | 367.15207 | 3.86 | Gingerenone B | 6 | 2.12383 | 0.764276441 | 2.80137E-07 |
| 421.16268_3.35 | 421.16268 | 3.35 | Lupinisol A | 7 | 2.67631 | 0.76060535 | 3.73079E-08 |
| 362.09576_0.65 | 362.09576 | 0.65 | (R)-S-Lactoylglutathione | 17 | 2.66441 | 0.756703163 | 2.94409E-07 |
| 509.2204_3.76 | 509.2204 | 3.76 | Deuteroporphyrin IX | 1 | 2.38487 | 0.754119057 | 2.06083E-08 |
| 101.02461_0.69 | 101.02461 | 0.69 | Acetoacetic acid | 1 | 2.81698 | 0.742029591 | 1.65527E-11 |
| 89.02476_0.67 | 89.02476 | 0.67 | Lactic acid | 3 | 4.0867 | 0.733627814 | 8.66273E-09 |
| 379.13473_4.28 | 379.13473 | 4.28 | xi-3-Hydroxy-5-phenylpentanoic acid O-beta-D-Glucopyranoside | 4 | 2.25434 | 0.732891306 | 1.84398E-08 |
| 483.1982_4.6 | 483.1982 | 4.6 | Hydrocortisone sodium succinate | 3 | 2.83637 | 0.703868374 | 1.29478E-09 |
| 225.06158_0.67 | 225.06158 | 0.67 | Glucoheptonic acid | 0 | 4.57064 | 0.699476198 | 1.90561E-10 |
| 563.13945_3.94 | 563.13945 | 3.94 | Kachimoside | 2 | 2.53096 | 0.698592737 | 8.22859E-09 |
| 457.17328_4.22 | 457.17328 | 4.22 | 3-Hydroxychavicol 1-[rhamnosyl-(1->6)-glucoside] | 3 | 4.38059 | 0.698121613 | 1.17349E-09 |
| 143.03549_0.68 | 143.03549 | 0.68 | (E)-hex-2-enedioic acid | 3 | 2.52868 | 0.695460909 | 1.33353E-09 |
| 167.01414_0.58 | 167.01414 | 0.58 | 3,4-Dihydroxy-2-butanone 4-phosphate | 19 | 2.08223 | 0.693896354 | 5.96681E-06 |
| 274.27274_5.84 | 274.27274 | 5.84 | C16 Sphinganine | 4 | 5.30035 | 0.692274524 | 0.009985674 |
| 102.03565_0.58 | 102.03565 | 0.58 | 4,5-Dihydro-2-methylthiazole | 15 | 2.28395 | 0.692238599 | 5.3701E-06 |
| 161.0458_0.68 | 161.0458 | 0.68 | 2-Hydroxyadipic acid | 1 | 4.00891 | 0.686680316 | 1.93727E-08 |
| 403.195_4.4 | 403.195 | 4.4 | 7α-(Thiomethyl)spironolactone sulfoxide | 0 | 1.68877 | 0.685863411 | 7.99682E-08 |
| 493.14535_4.21 | 493.14535 | 4.21 | 6'',6''-Dimethylpyraono[2'',3'':7,8]kaempferol 4'-methyl ether 3-rhamnoside | 9 | 2.13516 | 0.6826479 | 8.63108E-10 |
| 179.05637_0.67 | 179.05637 | 0.67 | α-D-Glucose | 1 | 8.68493 | 0.677701846 | 1.08985E-09 |
| 119.03384_0.67 | 119.03384 | 0.67 | 2,4-Dihydroxybutyric acid | 9 | 2.81483 | 0.671844722 | 1.0879E-09 |
| 173.03776_0.62 | 173.03776 | 0.62 | Quinoxaline-2-carboxylic acid | 12 | 2.60892 | 0.670661595 | 1.18355E-11 |
| **Var ID (Primary)** | **m/z** | **r.t(min)** | **metabolites** | **Δppm** | **VIP value** | **FC(T/CK)** | ***P*** |
| 121.02978_3.16 | 121.02978 | 3.16 | Benzoic acid | 2 | 5.82575 | 0.670190607 | 1.01723E-09 |
| 433.20687_3.45 | 433.20687 | 3.45 | Dexamethasone-21-Acetate | 12 | 2.75842 | 0.66890427 | 8.42153E-09 |
| 441.1783_2.93 | 441.1783 | 2.93 | Lusitanicoside | 3 | 2.0768 | 0.665515142 | 9.17891E-06 |
| 219.0241_0.63 | 219.0241 | 0.63 | α-D-Glucose | 11 | 2.06532 | 0.662967975 | 1.67913E-05 |
| 819.52307_13.85 | 819.52307 | 13.85 | PG(18:1(11Z)/22:6(4Z,7Z,10Z,13Z,16Z,19Z)) | 5 | 5.75787 | 0.651207172 | 1.05099E-06 |
| 117.01976_0.62 | 117.01976 | 0.62 | Succinic acid | 3 | 2.49893 | 0.634283553 | 1.31107E-05 |
| 203.04847_0.64 | 203.04847 | 0.64 | L-Methionine sulfoximine | 11 | 1.99525 | 0.626958771 | 1.62873E-06 |
| 461.22867_4.2 | 461.22867 | 4.2 | (-)-Jolkinol A | 8 | 1.96213 | 0.626232669 | 8.26556E-11 |
| 281.0658_0.64 | 281.0658 | 0.64 | Cysteinyl-Histidine | 7 | 2.72264 | 0.59915091 | 1.42334E-10 |
| 447.2239_4.6 | 447.2239 | 4.6 | Kenposide B | 0 | 4.20996 | 0.59109209 | 2.53962E-11 |
| 252.10715_0.9 | 252.10715 | 0.9 | 5'-Deoxyadenosine | 7 | 2.00199 | 0.573175439 | 1.71109E-09 |
| 361.18732_4.78 | 361.18732 | 4.78 | Fludrocortisone | 16 | 1.72818 | 0.55453083 | 1.16989E-11 |
| 448.22656_4.6 | 448.22656 | 4.6 | Cadabicine methyl ether | 5 | 2.08623 | 0.554000953 | 3.68207E-06 |
| 493.22806_4.56 | 493.22806 | 4.56 | Ustiloxin D | 4 | 3.86724 | 0.521522931 | 1.14607E-11 |
| 387.17693_5.46 | 387.17693 | 5.46 | Gingerenone B | 8 | 2.18542 | 0.480788666 | 0.005350363 |
| 97.99139_0.59 | 97.99139 | 0.59 | Amidosulfonic acid | 7 | 4.00462 | 0.457824991 | 0.004300009 |
| 215.03293_0.64 | 215.03293 | 0.64 | D-Mannose | 5 | 7.94666 | 0.416867349 | 1.83817E-13 |
| 217.02813_0.64 | 217.02813 | 0.64 | Aspartyl-Cysteine | 0 | 4.42418 | 0.408680003 | 1.99188E-15 |
| 549.15738_3.79 | 549.15738 | 3.79 | Pueraria glycoside | 5 | 2.25649 | 0.4071373 | 5.10393E-11 |
| 547.14573_3.86 | 547.14573 | 3.86 | Pueraria glycoside | 0 | 2.65349 | 0.347240957 | 4.87654E-11 |
