# Supplementary figures and images for: The Plant Growth-Promoting Fungus MF23 (Mycena sp.) Increases Production of Dendrobium officinale (Orchidaceae) by Affecting Nitrogen Uptake and NH4+ Assimilation
Source: Front Plant Sci. 2021 Jul 15;12:693561. doi: 10.3389/fpls.2021.693561 (PMC8451717; doi:10.3389/fpls.2021.693561)

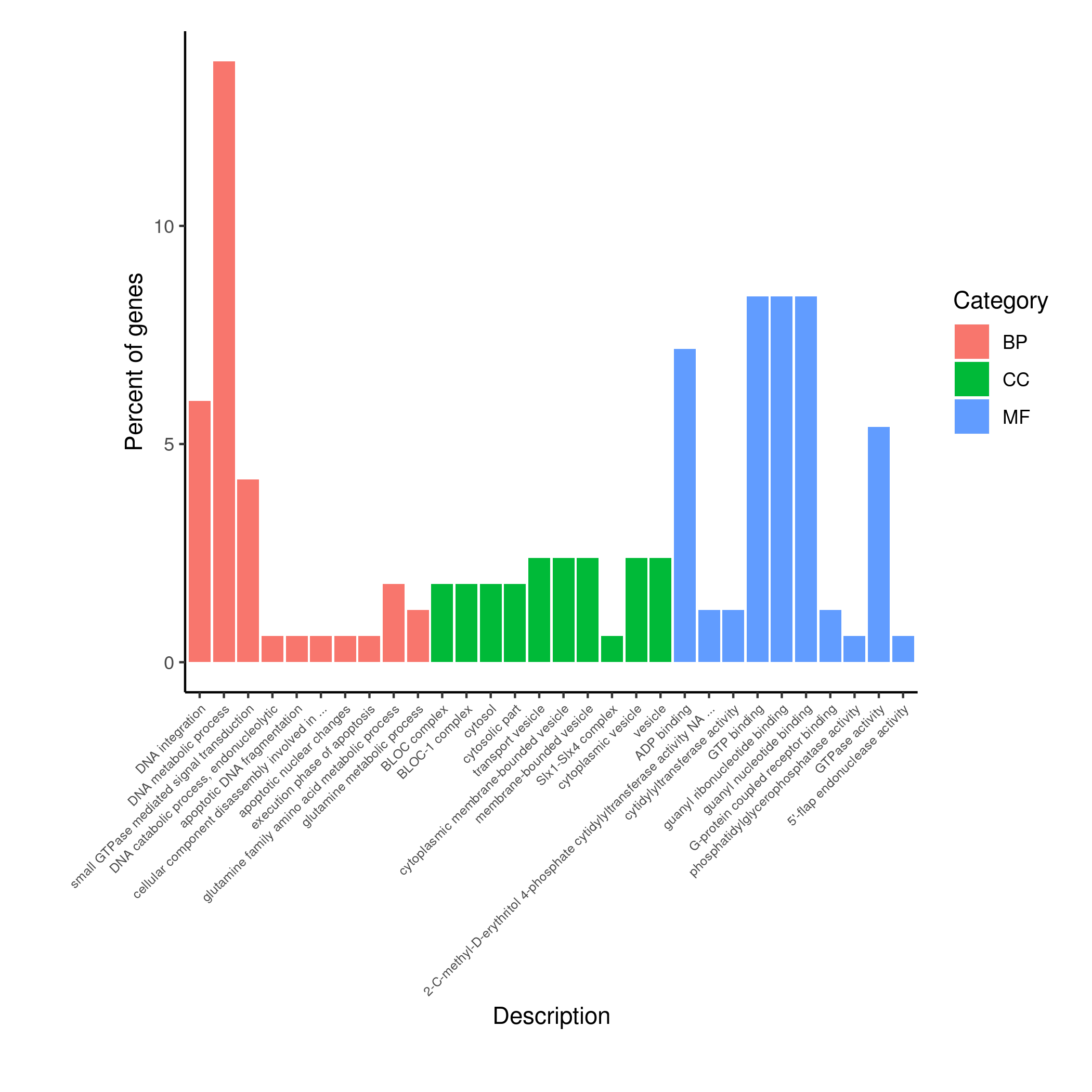

Supplement: Supplementary file 1 [file Data_Sheet_1.zip › Image_1.png]

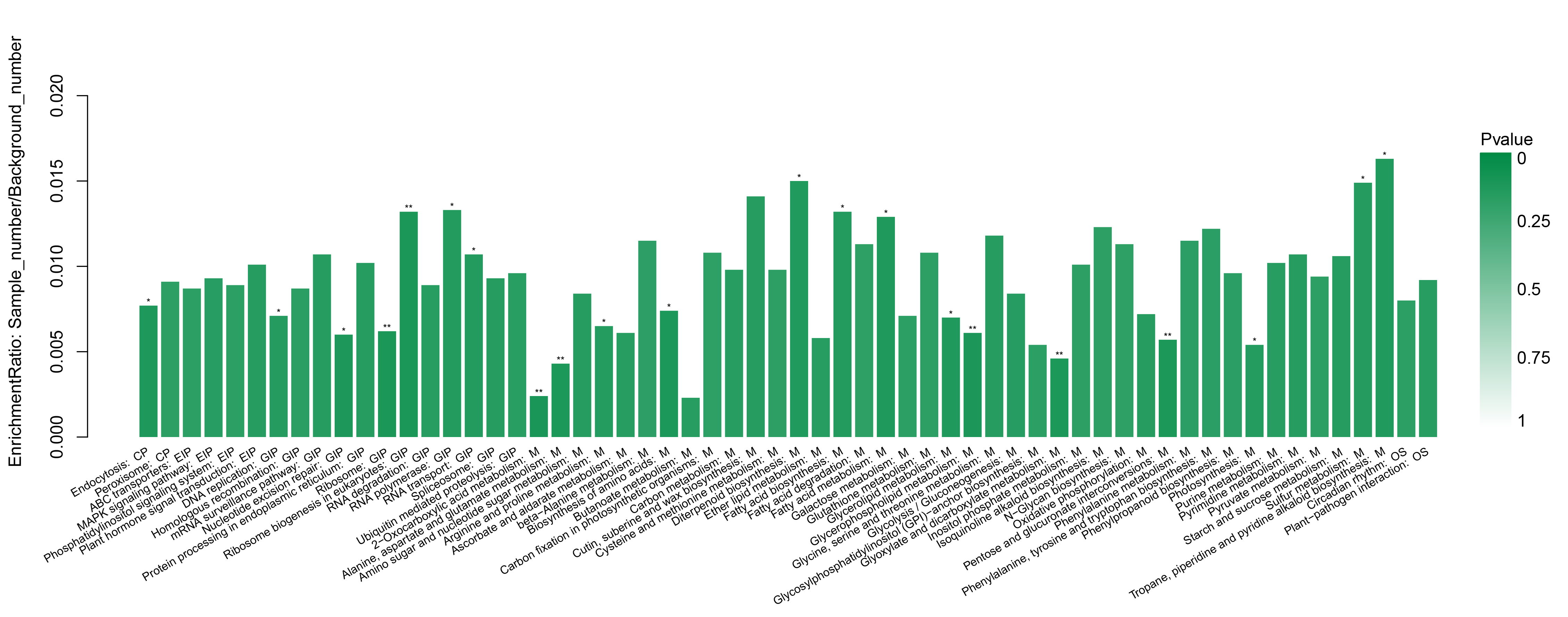

Supplement: Supplementary file 1 [file Data_Sheet_1.zip › Image_2.tif]

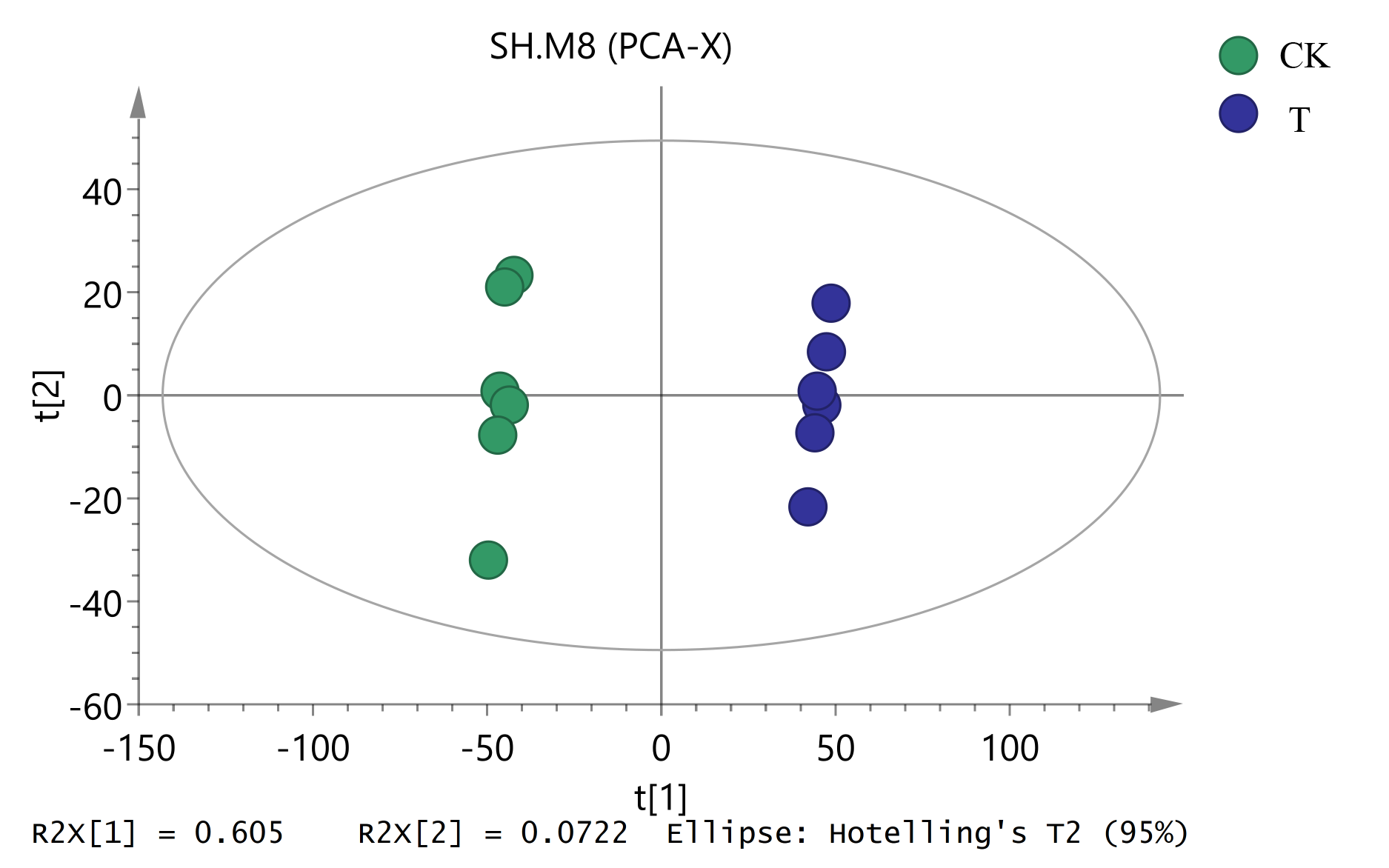

Supplement: Supplementary file 1 [file Data_Sheet_1.zip › Image_3.tif]

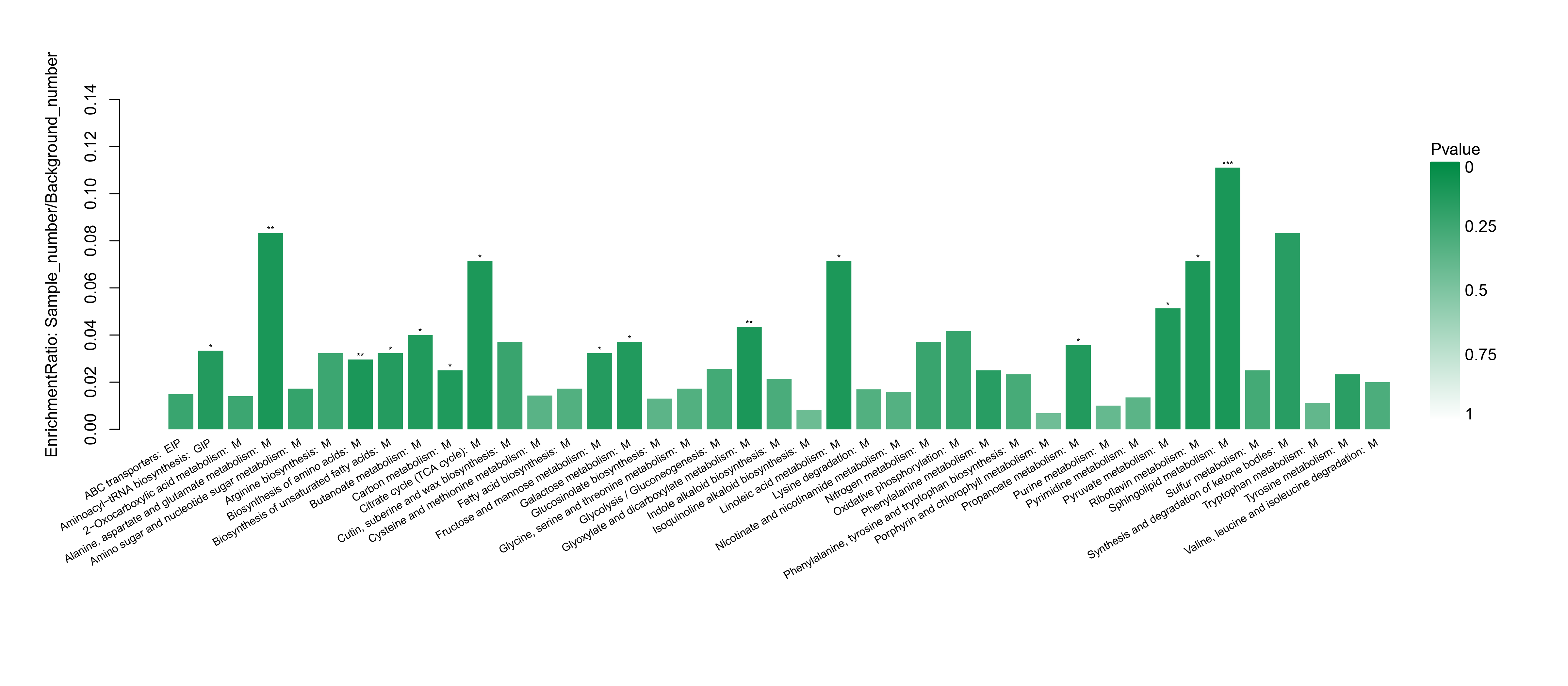

Supplement: Supplementary file 1 [file Data_Sheet_1.zip › Image_4.tif]

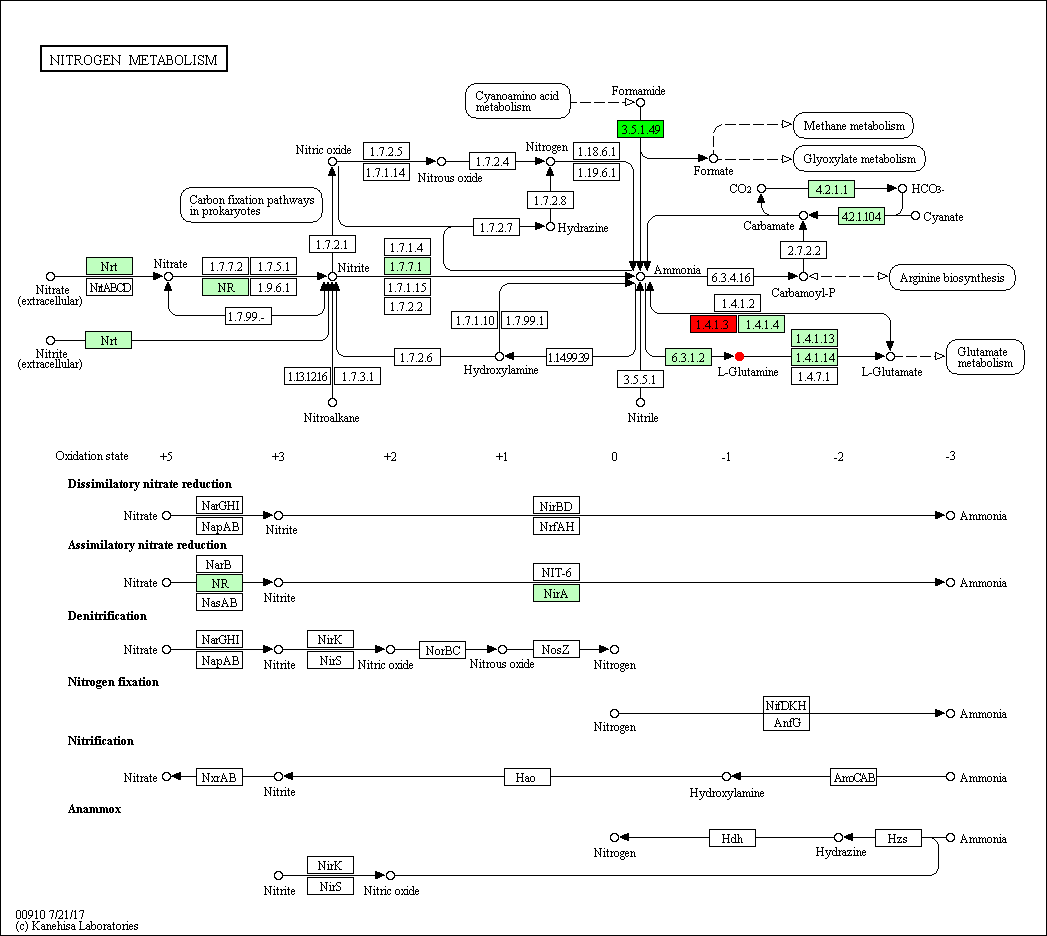

Supplement: Supplementary file 1 [file Data_Sheet_1.zip › Image_5.png]

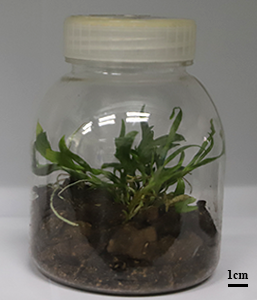

Supplement: Supplementary file 1 [file Data_Sheet_1.zip › Image_6.tif]
